# Genome Wide *In silico* Analysis of the Mismatch Repair Components of *Plasmodium falciparum* and Their Comparison with Human Host

**DOI:** 10.3389/fmicb.2017.00130

**Published:** 2017-02-09

**Authors:** Mohammed Tarique, Moaz Ahmad, Manish Chauhan, Renu Tuteja

**Affiliations:** Parasite Biology Group, International Centre for Genetic Engineering and BiotechnologyNew Delhi, India

**Keywords:** ATPase, DNA, helicase, malaria, mismatch repair, *Plasmodium falciparum*

## Abstract

Malaria a major parasitic infection globally particularly in tropical and sub-tropical regions of the world is responsible for about 198 million cases and estimated deaths due to this disease are about 0.6 million. The emergence of drug resistance in the malaria parasite is alarming and it is necessary to understand its underlying cause and molecular mechanisms. It has been established that drug resistant malaria parasites have defective mismatch repair (MMR) therefore it is essential to study this pathway and its components in detail. Recently a number of non-synonymous Single Nucleotide Polymorphisms have been reported in genes involved in MMR pathways. PfMLH is an endonuclease essential to restore the MMR in drug resistant strains of *Plasmodium falciparum*. Considering all these facts about the role of MMR in emergence of drug resistant parasite, in this manuscript we report a genome wide analysis of the components of the MMR pathway such as MLH, Pms1, MSH2-1, MSH2-2, MSH6, and UvrD using *in silico* bioinformatics based approaches. The phylogenetic analysis revealed evolutionary closeness with the MMR components of various organisms. It is noteworthy that *P. falciparum* contains two homologs of MSH2, which are located on different chromosomes. The structural modeling of these components showed their similarity with the human/yeast MMR components. The docking studies reveal that PfUvrD and PfMLH interact with each other. The *in silico* identification of interacting partners of the major MMR components identified numerous *P. falciparum* specific proteins. In line with our previous studies the present study will also contribute significantly to understand the MMR pathway of malaria parasite.

## Introduction

Human malaria is caused by different species of Plasmodium, an obligate intracellular parasite which requires two hosts to complete its life cycle, i.e., arthropod vector for sexual life cycle and human host for asexual life cycle (liver and blood stages) (Tuteja, 2007, 2010; Ahmad and Tuteja, 2014). Malaria remains a significant source of morbidity and mortality worldwide and is responsible for ∼0.66 million human deaths globally but recently death toll has slightly declined to ∼0.58 million (World Health Organization, 2011, 2015). Malaria infection rates are high in the tropical and subtropical regions of the developing world particularly in rural regions. *Plasmodium falciparum* is prone to immune evasion that is due to the antigenic variation contributed by the multicopy var gene family. *P. falciparum* erythrocyte membrane protein 1 (PfEMP1), encoded by this multicopy var gene family expressed on the surface of infected red blood cells, plays an important role in this immune evasion. The *var* gene family is highly polymorphic and this polymorphism occurs by recombination during mitosis and it has been reported that under drug pressure the parasite becomes hyper mutated (Bopp et al., 2013; Claessens et al., 2014; Brown et al., 2015). The efforts to identify potential drug targets and development of suitable drug(s) are of particular concern. There is an imperative necessity to explore the underlying causes of the emergence of drug resistance in the parasite, as well as development of drugs to treat the drug resistant malaria. It has been reported that defective mismatch repair (MMR) pathway is responsible for drug resistance in malaria parasite (Castellini et al., 2011) therefore MMR proteins of malaria parasite have gained attention for their characterization and role in drug resistance. It has been reported that human malaria parasite has unusual MMR pathway as compared to other eukaryotes (Bethke et al., 2007; Ahmad and Tuteja, 2014).

Mismatch repair is very specific DNA repair process responsible for correcting the mismatched DNA bases that occur during DNA metabolic processes. In addition, it also affects diverse cellular processes including DNA damage signaling, apoptosis, mitotic and meiotic recombination, class-switch recombination, somatic hyper mutation and triplet expansion (Cascalho et al., 1998; Song et al., 2010; Ahmad and Tuteja, 2014). Thus, DNA MMR contributes significantly to maintain the genome stability. MMR has been extensively studied in prokaryotic organisms like *Escherichia coli* as well as in higher organisms including human. *E. coli* MMR pathway is methyl-directed MMR pathway and serves as an excellent model for comparison with MMR machinery of higher organisms. *E. coli* MMR machinery is DNA strands specific and efficiently distinguishes the newly synthesized strand from mother stand by utilizing the absence of methylation in the new strand. The bacterial DNA MMR pathway requires a number of proteins to complete the repair in coordinated fashion. MMR process can be divided into three coupled stages: recognition-initiation, excision and repair DNA synthesis (Song et al., 2010). MutS homodimer initially serves to recognize mismatched bases then MutL-MutS-DNA complex activates the MutH endonuclease function (Fukui, 2010). Thus MutS, MutL, and MutH recognize mismatches in a coordinated fashion to initiate incision process to create a strand break in unmethylated (new) DNA strand (Song et al., 2010). MutH endonuclease is very specific to nick the unmethylated strand after recognition of mismatch and it nicks at a hemimethylated specific restriction site (GATC site) which provides an initiating point of the MMR process (Morita et al., 2010). One of the four exonucleases (Exo1, Exo VII, Exo X, or RecJ) along with UvrD helicase (DNA-helicase II) performs the 5′–3′ or 3′–5′ excision from the strand break. Finally, DNA polymerase III holoenzyme catalyzes the repair DNA synthesis and gap is sealed by DNA ligase (Song et al., 2010; Ahmad et al., 2012). MutH-less bacteria possess somewhat different mechanism for MMR due to the lack of MutH endonuclease and show some characteristic of the eukaryotic MMR pathway. Human MutL homolog contains a metal-binding motif towards C-terminal domain and exhibits endonuclease activity (Kadyrov et al., 2006). The endonuclease activity of MutL has been reported to be essential for the *Thermus thermophilus* DNA MMR (Fukui et al., 2008). Various studies have shown that certain MMR proteins in both prokaryotes and eukaryotes are common while there are some components specific to either prokaryotic or eukaryotic MMR pathway (Li, 2008). But it is noteworthy that prokaryotic and eukaryotic MMR machinery utilize similar basic mechanism, which is bidirectional and strand specific like eukaryotic MMR utilizes pre-existing nick instead of hemimethylation as in *E. coli* (Drummond et al., 1996; Yuan et al., 2009).

All the components of the eukaryotic MMR pathway have been identified and studied in detail. MMR components include homologs of MutL/MLH, MutS/MSH, EXO1, RPA, DNA polymerase δ, RFC, PCNA, and HMGB1 (Lo et al., 2005; Kunkel and Erie, 2005; Zhang et al., 2005; Song et al., 2010). *E. coli* genome contains only one MutS and one MutL but eukaryotes possess variable forms of MutL; MutLα/MLH1-PMS2 (MLH1-PMS1 in yeast), MutLβ/MLH1-PMS1 (MLH1–MLH2 in yeast) and MutLγ/MLH1–MLH3. Three forms of MutS heterodimers are known as MutSα (dimer of MSH2 and MSH6), MutSβ (dimer of MSH2 and MSH3) and third dimer is made up of MSH4 and MSH5 (Kunkel and Erie, 2005; Li, 2008; Song et al., 2010). MutSα recognizes both mismatches and small insertion deletion loop (IDLs) while MutLβ and MutLγ participate in recognition/repairing IDLs (Harfe and Jinks-Robertson, 2000; Song et al., 2010). In eukaryotic MMR pathway, MutL*α* is responsible for strand discrimination by incising the discontinuous strand (Kadyrov et al., 2006; Morita et al., 2010). Thus, endonuclease activity of MutLα is crucial in MMR and it is involved in the termination of non-specific excision after mismatch removal (Zhang et al., 2005; Kadyrov et al., 2006). MutH and UvrD homologs are not present in eukaryotes but an UvrD like protein Srs2 has been found critical for recombinational repair in yeast (Saponaro et al., 2010).

Mismatch repair machinery of malaria parasite has not been well studied but there are some reports on biochemical studies of few components such as MLH and UvrD (Ahmad et al., 2012; Tarique et al., 2012). Previous studies have shown that MMR machinery of *P. falciparum* is unusual as compared to the *E. coli* or eukaryotic MMR machinery. Similar to the prokaryotic system, *P. falciparum* contains an UvrD helicase which possesses all the characteristic motifs (Shankar and Tuteja, 2008). Previously we have reported the detailed characterization of MLH and UvrD helicase from *P. falciparum* and our studies show that PfUvrD and PfMLH interact and regulate each other’s biochemical activities (Shankar and Tuteja, 2008; Ahmad et al., 2012). *P. falciparum* contains UvrD helicase and lacks MutH similar to the MutH-less bacteria (Morita et al., 2010). It was reported previously that MLH (or MLH1) is required for the MMR machinery in both drug sensitive (3D7) and drug resistant strains (W2) of *P*. *falciparum* (Lee et al., 2014). Out of the two MutL homologs of *P. falciparum* MLH contains the complete MutL domain as compared to Pms1 (Shankar and Tuteja, 2008; Tarique et al., 2012). The biochemical characterization of *P. falciparum* MLH showed that it is active as endonuclease and also contains ATPase activity (Tarique et al., 2012).

Very little is known about mechanistic events of DNA repair mechanisms including MMR machinery in *P. falciparum* but the availability of its genome sequence has facilitated the comparative studies with human host and other organisms. Here we report the detailed *in silico* characterization of MMR components of *P. falciparum* and their comparison with the MMR machinery of human host. Therefore, this study will help to understand the MMR pathway of malaria parasite.

## Materials and Methods

All the sequences of the components of the MMR machinery (such as MutL, Pms1, MSH2-1, MSH2-2, and MSH6) from human were retrieved from the genome database NCBI^1^ and BLASTp analysis was done to identify the homologs in *Plasmodium falciparum*. These *P. falciparum* homologs sequences were retrieved from PlasmoDB^2^ and were used to fetch the homologs of other Plasmodium species. The FASTA formats of the retrieved sequences were used for further analysis. These retrieved sequences were also used for identifying the homologs in organisms other than human and Plasmodium such as *Mus muluscus, Arabidopsis thaliana, Saccharomyces cerevisiae*, *and Trypanosoma cruzi*. The retrieved sequences were *in silico* studied and various domains were manually assigned and confirmed by using Pfam, Prosite, SMART, PANTHER, etc., and integrated software, InterProScan (Quevillon et al., 2005). Similar to the previous reports (Tuteja, 2010; Tajedin et al., 2015), multiple-sequence alignment was done by using ClustalW^3^ and Clustal omega^4^ and conserved motifs were identified manually as well as using Pfam and InterProScan software. Phylogenetic tree analysis was done by using Phylogeny.fr online available server using MUSCLE for alignment and G blocks (v0.91b) for curation of alignment to remove uncertain regions due to gaps and poor alignment. PhyMl program (v3.1/3.0 aLRT) was used to reconstruct tree using maximum likelihood method and final editing and graphical presentation was done using TreeDyn (v198.3) (Castresana, 2000; Guindon et al., 2003, 2010; Edgar, 2004; Anisimova and Gascuel, 2006; Chevenet et al., 2006; Dereeper et al., 2008, 2010). The ribbon diagram of the template and the predicted structure of PfPms1, PfMSH2-1, PfMSH2-2, and PfMSH6 were prepared using SWISS-MODEL workspace server (Arnold et al., 2006) and images were superimposed for the analysis. Molecular graphic images were produced using the UCSF Chimera package^5^ from the Resource for Biocomputing, Visualization, and Informatics (supported by NIH P41 RR-01081) (Pettersen et al., 2004). To study stereochemical property of the predicted model we used prochek module of pdbsum server and Ramachandran plots were also generated using pdbsum to check the correctness of the predicted models^6^.

The protein–protein interaction study was also performed using amino acid sequences of MLH, PMS1, MSH2-1, MSH2-2, MSH6, and UvrD of *P. falciparum* and the multi protein complexes were analyzed using string software version 10 (Jensen et al., 2009; Franceschini et al., 2013; Szklarczyk et al., 2015) using high confidence parameters (0.9) and filtering number of interacting partners to ten. Protein sequences of *P. falciparum* MLH, Pms1. MSH2-1, MSH2-2, MSH6, and UvrD were used in STRING db v10 software which predicts the interactions of the input protein sequence on the basis of different prediction based networks. The interactions include direct (physical) and indirect (functional) associations. Multiple sources have been used to predict these interactions such as primary databases provide known experimental interactions and manually curated databases provide pathway knowledge. Some interactions are also predicted de novo using genomic information and interactions observed in one organism are systematically transferred to the other organisms also. All sources of interaction evidence are benchmarked and calibrated against previous knowledge, using the high-level functional groupings provided by the manually curated Kyoto Encyclopedia of Genes and Genomes (KEGG) pathway maps.

Gene neighborhood, gene fusion, phylogenetic profile; sequence based methods such as Interlogs and phylogenetic tree method; structure based predictions including structure docking methods; and domain based methods were used via String to predict interaction between proteins. As all of these are computational based prediction methods they have certain limitations with them. The benchmark or test set particular software uses are general in nature, like if the protein homolog is present in different organism and it is characterized in a pathway of one organism then the software will be biased towards assigning the same interacting partners for the homolog in another organism. Genomic methods of prediction predict interactions with high specificity such as for gene fusion specificity is 72% (Yanai et al., 2001).

The amino acid sequences of PfMLH and PfUvrD were uploaded on SWISS MODEL server and program was used to select suitable homology based template. The structural models were obtained using these templates and Pdb files of the predicted models were further used for molecular docking. Docking was performed by online server Patch Dock^7^, a molecular docking algorithm based on shape complementarity and the top scoring result was selected for structural analysis (Duhovny et al., 2002; Schneidman-Duhovny et al., 2005). Both PDB files of PfUvrD and PfMLH were uploaded into the Patch dock server for protein–protein docking simulation. The run time of Patch dock for two input proteins (PfUvrD and PfMLH) were <10 min on a single 1.0 GHz PC processor under the linux operating system. Scoring is based on a combination of geometric fit and atomic de- solvation energy. The residual interactions between the expected three dimensional model of PfMLH and PfUvrD were visualized by Discovery studio 3.5.

## Results and Discussion

In this study, we have reported the *in silico* analysis of all the major components of MMR machinery of malaria parasite *P. falciparum* and their comparative analysis with the components of the MMR pathway of human host and/or yeast. The major components of the MMR pathway of malaria parasite are expected to include MLH1, Pms1, MSH2-1, MSH2-2, MSH6, and UvrD helicase. There are some differences in the major MMR components such as MSH3 is lacking in the parasite whereas UvrD is not present in the human host. Despite being a eukaryote, the malaria parasite shares some of its components with the prokaryotic repair system such as UvrD in MutH less bacteria. Except MLH1 other components of the parasite show low similarity with the host due to *P. falciparum* specific insertions and asparagine rich region insertions.

Previously we have reported the characterization of *P. falciparum* MMR component MLH which was identified as ssDNA dependent ATPase and it possesses characteristic endonuclease activity (Tarique et al., 2012). Similarly UvrD helicase has also been characterized and the biochemical studies showed that PfUvrD exhibits 3′–5′ direction specific DNA helicase activity and it interacts with MLH (Ahmad et al., 2012). The detailed *in silico* comparative analysis of other components of *P. falciparum* MMR pathway such as MLH1, Pms1, MSH2-1, MSH2-2, MSH6, and UvrD has been done and presented here. Multiple sequence alignments of *P. falciparum* with yeast, human and with other Plasmodium species are also presented as Supplementary Figures.

### MutL Homolog (MLH)

MutL Homolog (MLH) is one of the highly conserved components of MMR pathway present in both prokaryotes and eukaryotes. Prokaryotic MLH is encoded as a single polypeptide which forms functionally active homodimer but in case of eukaryotes it is encoded as several different polypeptides forming functional heterodimers like MutLα (Mlh1 and Pms1) in yeast and MLH1 and PMS2 in humans; MutLβ (MLH1-PMS1) and MutLγ (MLH1–MLH3) (Cannavo et al., 2005). MutL homologs consist of two conserved structural domains, one highly conserved N-terminal domain and non-conserved C-terminal domain joined through a linker region of variable length. MutLα proteins have weak ATPase activity which is stimulated in the presence of DNA. The N-terminal domain is subdivided into two subdomains, first GHKL (Gyrase Hsp90, Histidine kinase, MutL) family domain having four conserved motifs and second DNA binding domain (Ban and Yang, 1998). There is lack of conservation in C-terminal domain of MutLα that is thought to be functionally inert but few reports showed weak endonuclease activity of MutLα which is enhanced upon addition of other components such as MutSα, ATP, RFC, and PCNA (Kadyrov et al., 2006).

**FIGURE 1:**
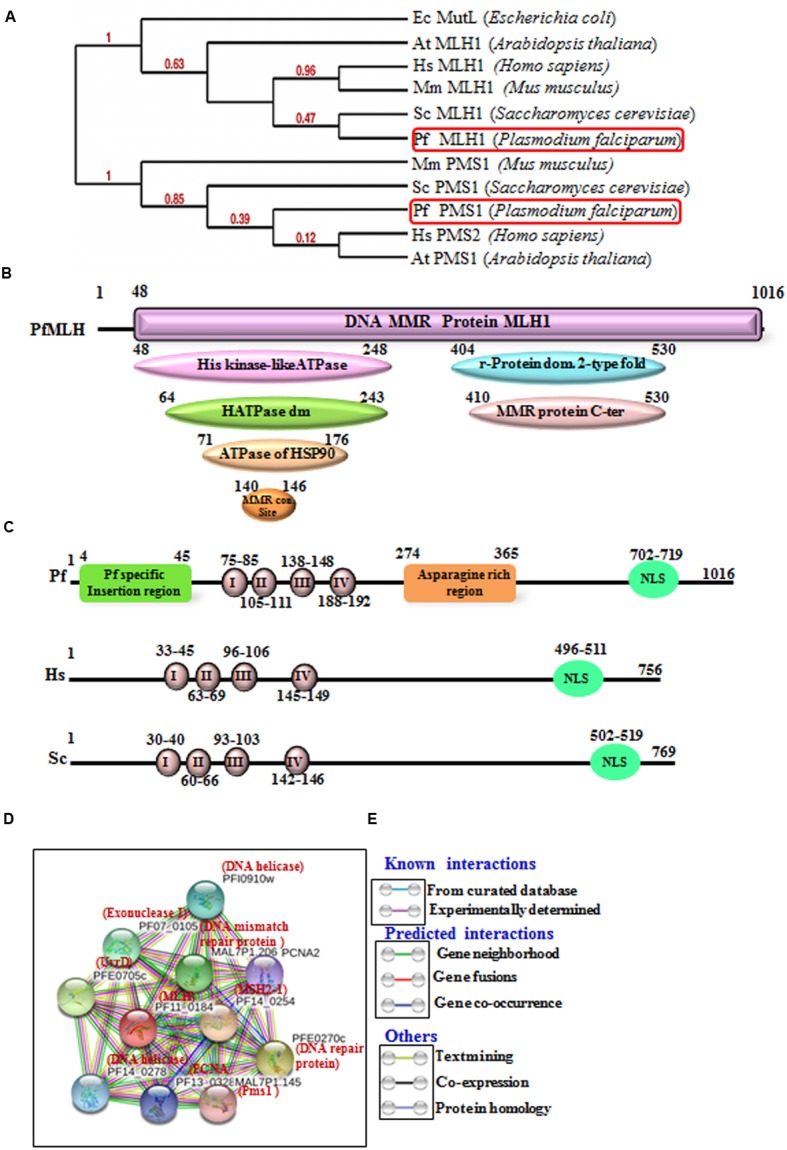
**(A)** The phylogenetic tree was obtained using the maximum likelihood method. The numbers in the tree show the distance between different sequences. The tree is drawn to scale with branch lengths (next to each branch) in the same units as those of the evolutionary distances used to infer the phylogenetic tree. **(B)** Detailed graphical organization of *P. flaciparum* MutL Homolog (MLH) domains with their specific position in the protein obtained by *in silico* tool/InterProScan; **(C)** Schematic comparison of conserved motifs of *P. flaciparum* MLH with human and Yeast. **(D)** Interacting protein prediction of PfMLH using STRING v10 database. **(E)** Explanation of interactions shown in **(D)**.

There are two homologs of MutL present in the *P. falciparum* genome MLH1 (PF11_0184) and Pms1 homolog (MAL7P1.145) (Supplementary Table S1) (Shankar and Tuteja, 2008) and the BLAST-P analysis of PfMLH amino acid sequence showed ∼50% identity with the human MLH1 (Supplementary Table S1). The protein sequence of MLH was aligned with the MLH/MutL sequence of yeast and human and conserved motifs are boxed in red color (Supplementary Figure S1A). The results clearly indicate that PfMLH contains all the characteristic motifs including DNA binding motif (Supplementary Figure S1A). Similarly, PfMLH sequence was aligned with the homologs in other species of Plasmodium using multiple sequence alignment analysis. The results show that MLH is highly conserved and it possesses all the characteristic motifs in other species of Plasmodium also (Supplementary Figure S1B). The amino acid sequence of PfMLH was also used to perform the phylogenetic analysis with the MLH, Pms1, MutL of human, yeast, bacteria, and other organisms. PfMLH shows closeness to the yeast MLH1 because the bootstrap value is 0.47 (**Figure 1A**). Similarly, the study of MLH amino acid sequences with various *in silico* tool/InterProScan revealed that MLH possesses its characteristic domains such as evolutionarily conserved N-terminal having Histidine kinase like ATPase domain also known as GHKL domain. This domain is present in ATP binding proteins like histidine kinase, DNA gyrase B, Hsp90, toposiomerase, phytochromatin-like ATPases and DNA mismatch proteins. MLH also contains DNA mismatch repair conserved site (G-F-R-G-E-[AG]-L) which is present in proteins involved in MMR in various organisms (Prodromou et al., 1997; Dutta and Inouye, 2000; Bellon et al., 2004; Immormino et al., 2004; Roe et al., 2004; Wright et al., 2004). The C-terminal domain contains ribosomal protein S5 domain 2 type fold which is present in numerous DNA/RNA binding proteins and it is also present in GHMP kinase family of proteins (Guarne et al., 2001) (**Figure 1B**). The sequence analysis of PfMLH, human MLH and yeast MLH revealed that the N-terminal is conserved in all three proteins but the position of ATPase domains and the conserved motifs are different (**Figure 1C**).

In order to identify the interacting proteins of the PfMLH, *in silico* approach STRING v10 with the parameters described in materials and methods was used (Jensen et al., 2009; Franceschini et al., 2013; Szklarczyk et al., 2015). String analysis provides a critical evaluation and integration of protein–protein interactions, including direct (physical) as well as indirect (functional) associations. The line color indicates the type of interaction evidence and these interactions are further categorized as known interactions (from curated database and experimentally determined); predicted interactions (from gene neighborhood, gene fusions and gene co-occurrence) and others (from text mining, co-expression and protein homology modeling). The results show that PfMLH interacts with Pms1, PfMSH2, PCNA, UvrD (**Figures 1D,E**) and some other proteins associated with the mismatch repair. Thus, these data indicate that the identified PfMLH possesses expected interacting partners along with some PfMLH specific interacting proteins (Supplementary Table S2).

### Postmeotic Segregation Increased 1 (Pms1)

Pms1 (*S. cerevisiae*) and Pms2 in humans in conjunction with MLH1 constitute MutLα heterocomplex and the major MutL functionality is contributed by this heterodimer complex. The repair is initiated due to binding of MutS to mismatched bases which in turn recruits MutLα. Due to the recruitment of MutS-MutLα heterocomplex on mismatches, the assembly of RFC and PCNA occurs which in turn activates the endonuclease activity of Pms2. It then creates a nick near the mismatch and forms a place for exonuclease Exo1 to act on the nick and degrade the strand having mismatched base (Constantin et al., 2005). During the process of DNA repair, Pms2 also acts as signal for DNA damage signaling which halts cell cycle and can progress to cell death via apoptosis if damage is irreparable (Shimodaira et al., 2003).

**FIGURE 2:**
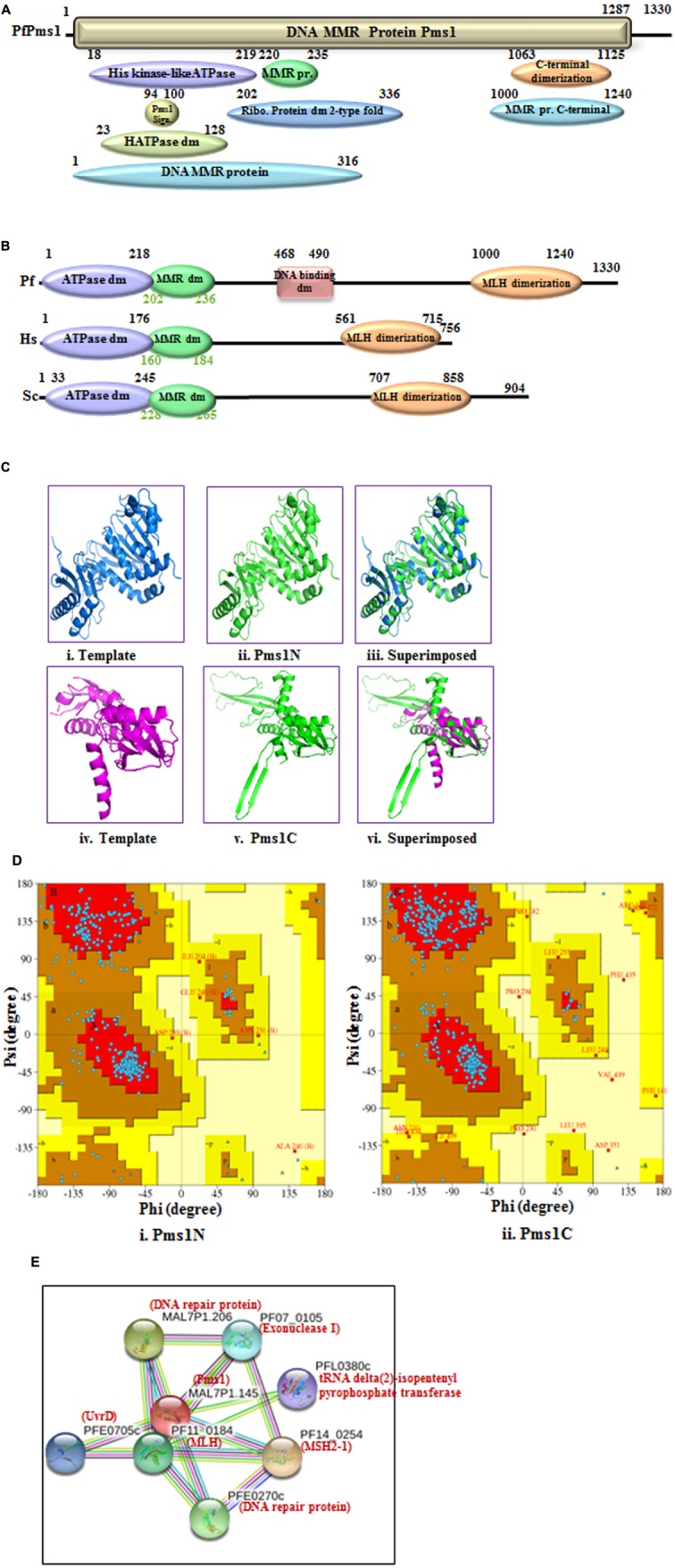
**Detailed graphical organization of *P. flaciparum* Pms1 domains with their specific position. (B)** Schematic comparison of aligned sequence of *P. flaciparum* Pms1 with humans and Yeast; **(C)** Structural modeling of PfPms1. The PfPms1 sequence was submitted to the Swiss model server and the structure was obtained. The molecular graphic images were produced using the UCSF Chimera package from the resource for Biocomputing, Visualization, and Informatics (http://www.cgl.ucsf.edu/chimera) at the University of California, San Francisco (supported by NIH P41 RR-01081). **(i)** template (3H4LB) for Pms1-N terminal; **(ii)** Pms1-N terminal **(iii)** superimposed image; **(vi)** template (4E4W) for Pms1-C terminal; **(v)** PfPms-C terminal; **(vi)** Superimposed image. **(D)** **(i,ii)** Ramachandaran plot for N and C-terminal predicted structures. **(E)** Interacting protein prediction of PfPms1.

PfPms1 is the second homolog of MutL similar to the other eukaryotic Pms1 and it showed ∼30% identity with human Pms2 (Supplementary Table S1). The results of alignment show that PfPms1 contains all the characteristic motifs like ATPase domain, MMR domain and DNA binding domain (Supplementary Figure S2A). Similarly, Pms1 sequences of other species of Plasmodium were used for multiple sequence analysis and the results reveal that Pms1 is also conserved among all the other species of Plasmodium (Supplementary Figure S2B). Along with MLH, we also performed the phylogenetic analysis of PfPms1 with the MLH, Pms1 and MutL of human, yeast, bacteria and other organisms. The results show that PfPms1 is closer to the human and yeast PfPms1 (**Figure 1A**). The results of InterProScan revealed that PfPms1 possesses characteristic motifs of Pms1 (**Figure 2A**). In addition to domains present in MLH, PfPms1 also has C-terminal dimerization domain which is not present in MLH1 and it lacks asparagine rich region insertion that is present in PfMLH1 (**Figure 2B**). The multiple sequence alignment of PfPms1 with yeast Pms1 and human Pms2 revealed the similarity of conserved motifs but PfPms1 also contains additional signature domains such as DNA binding domain which is absent in yeast and human homologs (**Figure 2B**).

The complete amino acid sequence of PfPms1 was submitted to the swiss model homology-modeling server^8^ for structure modeling (Arnold et al., 2006). The structural modeling of PfPms1 was done using the known crystal structure of the yeast Pms1 as template. The modeling of N-terminal (5 to 341 amino acids) of PfPms1 was done (**Figure 2Cii**) using the N-terminal domain of *Saccharomyces cerevisiae* Pms1 as template (3H4LB) (**Figure 2Ci**) while its C-terminal (917 to 1219 amino acids) was modeled (**Figure 2Cv**) using C-terminal domain of the *S. cerevisiae* MutL alpha (MLH1/PMS1) heterodimer (4E4W) (**Figure 2Civ**) as template. The results show that PfPms1N and C-terminal models partially superimpose on their template (**Figure 2Ciii,vi**, respectively). Although, their structure cannot be established on the basis of these data but at least it provides insight that the modeled structures show similarity with their template. The Ramachandran plot of PfPms1N indicates that 80.60% residues are in most favored region and provide evidence that the model is steriochemically stable (**Figure 2Di**). Similarly, the Ramachandran plot of PfPms1C indicates that 86.40% residues are in most favored region and the model is steriochemically stable (**Figure 2Dii**).

The interacting partners of PfPms1 were identified using STRING v10. The results show that PfPms1 might be interacting with several proteins expected to participate in the parasite MMR (**Figure 2E**) (Supplementary Table S2).

### MutS Homolog (MSH)

MutS is a conserved component of MMR throughout the course of evolution. In *E. coli* mutants of MutS show higher mutation frequencies of error misincorporations and have no effect of lethal alkylating agents (Karran and Marinus, 1982; Lahue et al., 1989). In yeast, human and other eukaryotes six MutS homolog (MSH)1-6 are present and these are responsible for DNA MMR in nucleus or in mitochondria. MutS complex has active role in recognition of DNA mismatches in the nucleus which is done by two polypeptide heterodimers MSH2/MSH6 (for base/base mismatches) and MSH2/MSH3 (for insertion/deletion loop outs). MutSα component helps in recruitment of MLH-PMS1 heterodimer and in this recruitment major contributors of MutSα homologs are MSH2-MSH6 heterodimer (Drotschmann et al., 2002). The human genome contains one MSH2 gene with two alternative spliced forms of MSH2 mRNA thus producing two isoforms of MSH2 protein known as MSH2-1 and MSH2-2 with difference of few amino acids at the N terminal. But in *P. falciparum* there are two MSH2 genes located at two different chromosomes. These will be described in following sections.

#### MSH2-1

**FIGURE 3:**
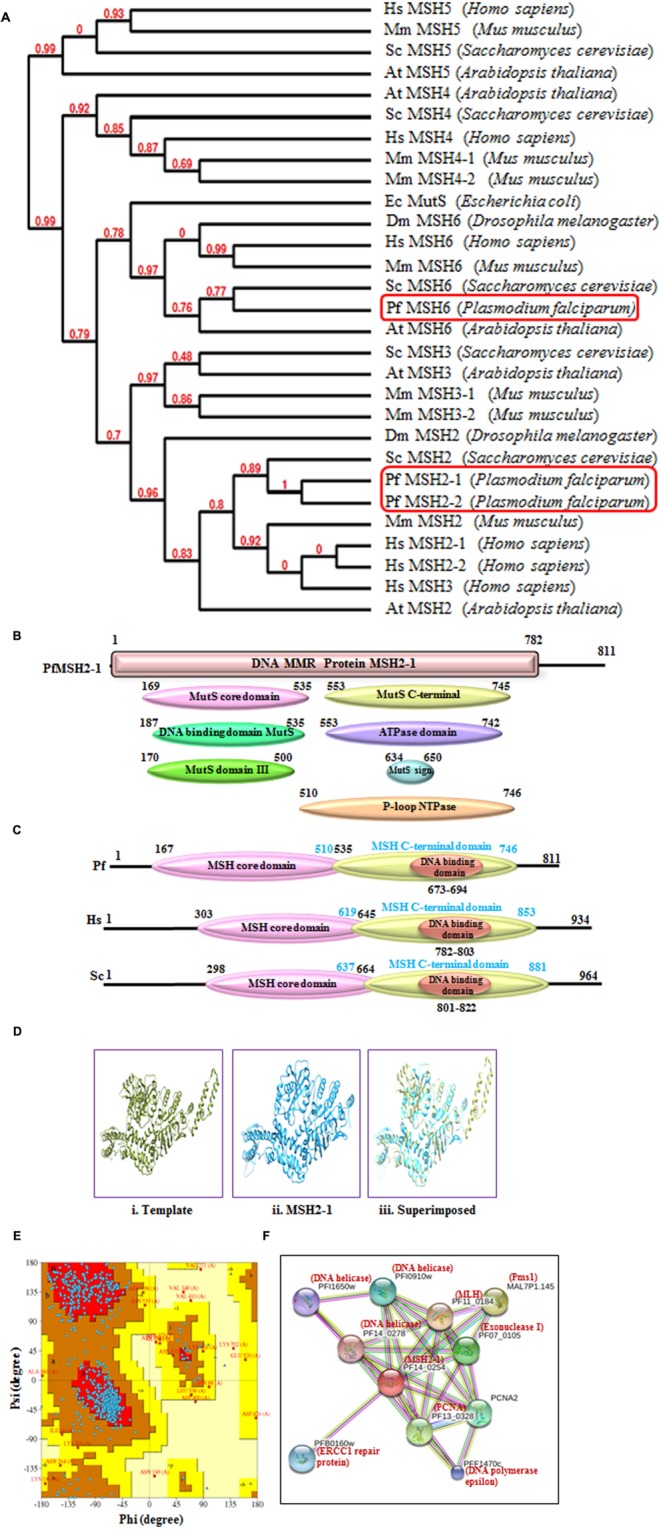
**(A)** The phylogenetic tree was obtained using the maximum likelihood method. The numbers in the tree show the distance between different sequences. The tree is drawn to scale with branch lengths (next to each branch) in the same units as those of the evolutionary distances used to infer the phylogenetic tree; **(B)** Detailed graphical organization of *P. flaciparum* MSH2-1 domains with their specific position. **(C)** Schematic comparison of aligned sequence of *P. flaciparum* MSH2-1 with humans and Yeast; **(D)** Structural modeling of PfMSH2-1. **(i)** Template is human MSH2-1 (PDB No. 3THWA), **(ii)** PfMSH2-1 **(iii)** superimposed image. **(E)** Ramachandaran Plot of PfMSH2-1 predicted structure. **(F)** Interacting protein prediction of PfMSH2-1.

*Plasmodium falciparum* contains three putative MSH and further characterization along with phylogenetic analysis revealed that these are MSH2-1, MSH2-2, and MSH6 proteins (**Figure 3A**). The phylogenetic analysis of PfMSH2-1 with MSH from variety of organisms’ shows that it is closer to the human and yeast MSH2 (**Figure 3A**). PfMSH2-1 showed ∼34% identity with human MSH2-1 (Supplementary Table S1). The InterProScan analysis revealed that PfMSH2-1 possesses all the characteristic domains (**Figure 3B**). The protein sequence alignment of PfMSH2-1 with the MSH2-1 sequence of yeast and human showed that PfMSH2-1 contains all the characteristic conserved motifs (Supplementary Figure S3A). Similarly, multiple sequence alignment of PfMSH2-1 with other Plasmodium species revealed that MSH2-1 is conserved among all the Plasmodium species (Supplementary Figure S3B). On the basis of multiple sequence alignment a schematic was prepared which shows the characteristic domains of MSH2-1 (**Figure 3C**). Structural modeling of PfMSH2-1 (2 to 750 amino acids) was done using the known crystal structure of the human MutS (3THWA) as the default template. The results reveal that the modeled structure of MSH2-1 is partially similar to the human MSH2-1 (**Figure 3Dii–iii**). The Ramachandaran plot of MSH2-1 indicates that 82.50% residues are in most favored region suggesting that the model is steriochemically stable (**Figure 3E**). The interaction study of PfMSH2-1 was also performed (**Figure 3F**) by using the amino acid sequences and String v10 software (Jensen et al., 2009; Franceschini et al., 2013; Szklarczyk et al., 2015). The results show that it interacts with various MMR components listed in Supplementary Table S2.

#### MSH2-2

**FIGURE 4:**
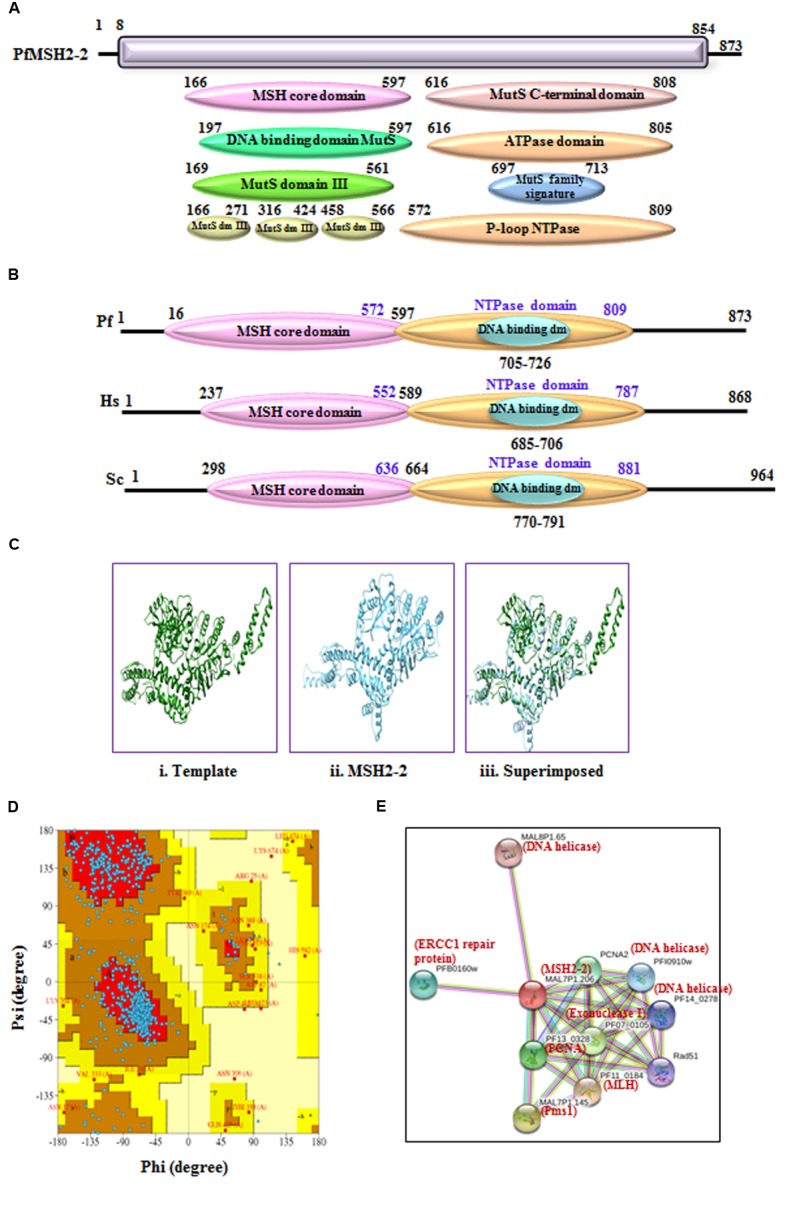
**(A)** Detailed graphical organization of *P. flaciparum* MSH2-2 domains with their specific position in the protein. **(B)** Schematic comparison of aligned sequence of *P. flaciparum* MSH2-2 with humans and Yeast; **(C)** Structural modeling of PfMSH2-2. (i) Template human MSH2 (PDB No. 3THWA) (ii) PfMSH2-2 (iii) Superimposed image. **(D)** Ramachandaran plot of PfMSH2-2 predicted structure. **(E)** Interacting protein prediction of PfMSH2-2.

The phylogenetic analysis of PfMSH2-2 with MSH from variety of organisms’ shows that it is closer to the human and yeast MSH2 (**Figure 3A**). PfMSH2-2 showed ∼33% identity with human MSH2-2 protein (Supplementary Table S1). The InterProScan analysis shows that PfMSH2-2 possesses all the characteristic domains of MSH2-2 (**Figures 4A,B**). The results of multiple sequence alignment of PfMSH2-2 with the MSH2-2 sequence of yeast and human suggest that PfMSH2-2 contains characteristic motifs of MSH2-2 (Supplementary Figure S4A). Similarly, the results of multiple sequence alignment of MSH2-2 sequence of other species of Plasmodium reveal that MSH2-2 is significantly conserved among other Plasmodium species also (Supplementary Figure S4B). The structural modeling of PfMSH2-2 (9 to 813 amino acids) was done using the known crystal structure of the human MutS (3THWA) as the template and the result shows partial similarity with the template (**Figure 4Cii,iii**). The analysis of the modeled structure of PfMSH2-2 with Ramachandran plot indicates that 81.40% residues are in most favored region which suggests that the model is steriochemicaly stable (**Figure 4D**). In order to identify the interacting proteins of PfMSH2-2, STRING v10 was used (Jensen et al., 2009; Franceschini et al., 2013) (**Figure 4E**). The results show that PfMSH2-2 interacts with various proteins related to the DNA repair pathway (Supplementary Table S2).

#### PfMSH6

**FIGURE 5:**
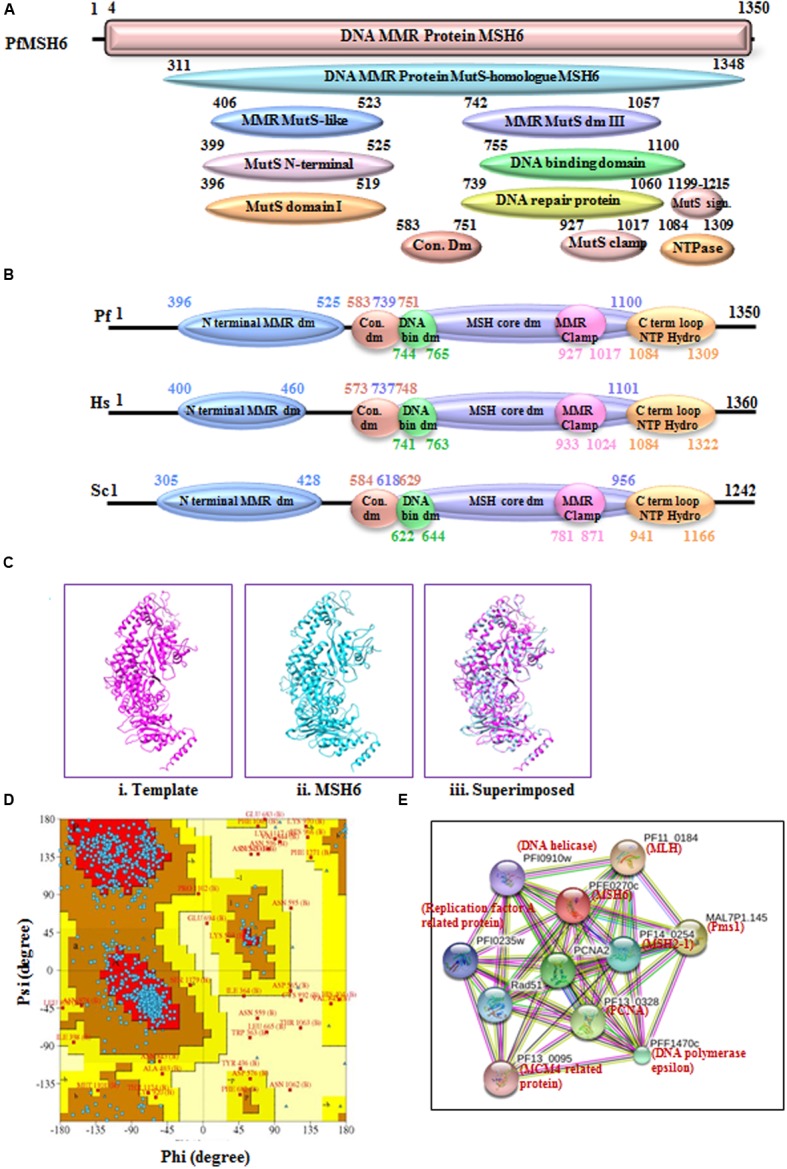
**(A)** Detailed graphical organization of *P. flaciparum* MSH6 domains with their specific position in the protein. **(B)** Schematic comparison of aligned sequence of *P. falciparum* MSH6 with humans and Yeast; **(C)** Structural modeling of PfMSH6. **(i)** Template human MSH6 (PDB No. 2O8EB), **(ii)** PfMSH6 **(iii)** superimposed image **(D)** Ramachandaran Plot of PfMSH6 predicted structure. **(E)** Interacting proteins prediction of PfMSH6.

MSH6 is another homolog of the MutS protein and the sequence analysis of PfMSH6 showed 36% identity with human MSH6 protein (Supplementary Table S1). The phylogenetic analysis of PfMSH6 with MSH from variety of organisms’ shows that it is closer to the yeast and plant MSH6 and shows divergence from MSH2-1 and MSH2-2 proteins (**Figure 3A**). The InterProScan analysis shows that PfMSH6 possesses all the characteristic conserved motifs of MSH6 (**Figures 5A,B**). The multiple sequence alignment of protein sequence of PfMSH6 with yeast and human sequences indicates that PfMSH6 contains all the characteristic motifs of MSH6 (Supplementary Figure S5A). Similarly, MSH6 in other species of Plasmodium is also significantly conserved (Supplementary Figure S5B). The complete sequence of PfMSH6 was submitted to the swissmodel homology-modeling server^9^ (Arnold et al., 2006) for molecular modeling. The structural modeling of PfMSH6 (351 to 1315 amino acids) was done using the known crystal structure of the human MutSα (MSH2/MSH6) (2O8EB) as the template. The ribbon diagram of PfMSH6 shows the predicted structure (**Figure 5Cii,iii**). The *in silico* analysis of the model of PfMSH6 using Ramachandaran plot revealed that 81.00% residues are in most favored region suggesting that the model is steriochemically stable (**Figure 5D**). The protein interaction study of PfMSH6 was done by using STRING v10 (Jensen et al., 2009; Franceschini et al., 2013; Szklarczyk et al., 2015) (**Figure 5E**) and the results show that it interacts with PCNA, MLH, and other MMR components (Supplementary Table S2).

### UvrD Helicase

**FIGURE 6:**
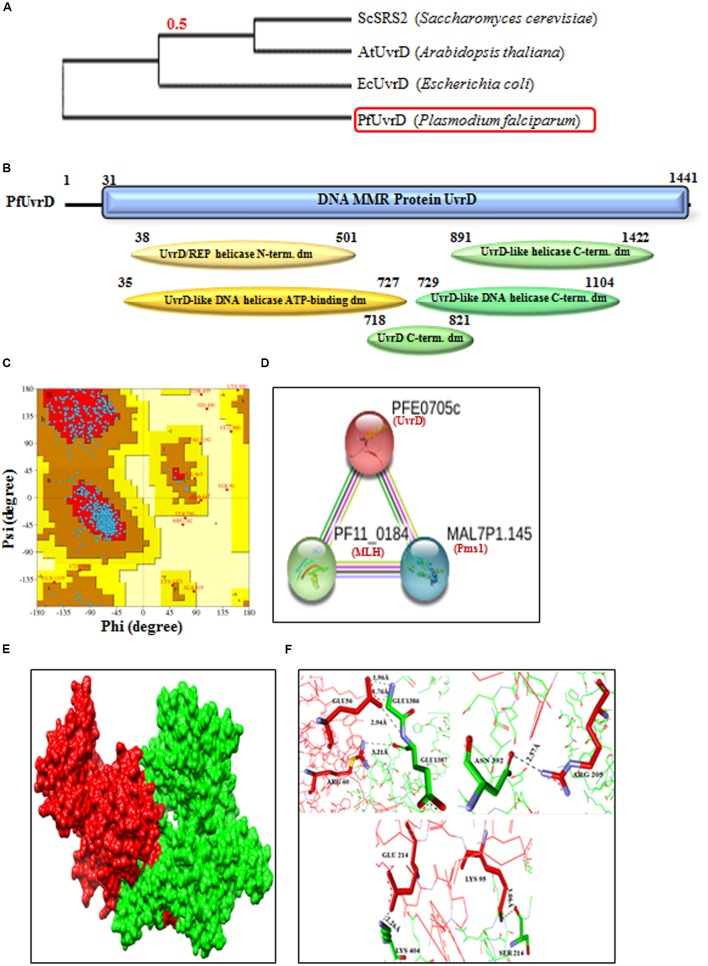
**(A)** The phylogenetic tree was obtained using the maximum likelihood method. The numbers in the tree show the distance between different sequences. The tree is drawn to scale with branch lengths (next to each branch) in the same units as those of the evolutionary distances used to infer the phylogenetic tree; **(B)** Detailed graphical organization of *P. flaciparum* UvrD domains with their specific position in the protein. **(C)** Ramachandaran Plot of PfUvrD predicted structure reported earlier (Ahmad et al., 2012). **(D)** Interacting proteins prediction of PfUvrD. **(E)** Molecular docking; Red is PfMLH and green is PfUvrD. **(F)** Interaction between the docking partners is through both polar and nonpolar interactions. Some of them (closest ones) have been depicted here.

Previously we have characterized the PfUvrD helicase and reported interesting findings about this protein. *P. falciparum* UvrD helicase is almost double in size as compared to the *E. coli* UvrD (∼82 kDa) (Ahmad et al., 2012) and possesses several characteristic domains towards its N-terminal region and some in the C-terminal region. The studies on UvrD from *E. coli* (Matson and Robertson, 2006) and Mycobacterium (Sinha et al., 2007, 2008) revealed that it is crucial component in the DNA repair mechanism of prokaryotes and is essential for the MMR and nucleotide excision repair (NER). The phylogenetic analysis of PfUvrD shows that it is closer to the *E. coli* UvrD and shows divergence from plant UvrD and yeast SRS2 proteins (**Figure 6A**). The *in silico* analysis of amino acid sequence of PfUvrD helicase revealed that it contains characteristic UvrD motifs (**Figure 6B**). Previously we have reported that all the Plasmodium species contain UvrD and *P. falciparum* contains the largest UvrD and this enzyme is significantly variable at the sequence and structural level among all the species of Plasmodium (Tuteja, 2013). In previous studies we have reported that PfUvrD contains DNA helicase and ssDNA-dependent ATPase activities (Ahmad et al., 2012). Previously we reported the modeled structure of PfUvrD and its comparative analysis with bacterial UvrD (Shankar and Tuteja, 2008; Ahmad et al., 2012). In this study, we have performed further analysis of modeled structure of PfUvrD with Ramachandran plot and the results reveal that 88.3% residues are in most favored region which show that model is steriochemicaly stable (**Figure 6C**). We have also reported that both MLH and UvrD interact with each other and to obtain further insight about the interacting proteins *in silico* study was performed using STRING v10. The results show that PfUvrD interacts with only two proteins MLH and Pms1 (**Figure 6D**) (Supplementary Table S2). The interaction between PfMLH and PfUvrD was analyzed further using docking. The results show that this interaction is through both polar and nonpolar residues. Some of them (closest ones) have been depicted in the figure. The results revealed that the residues Glu 214, Lys 95 and Arg 205 of PfMLH interact with Lys 404, Ser 214 and Asn 392 of PfUvrD, respectively, and these are present in the helicase N-terminal domain of PfUvrD (**Figures 6E,F**). These results also show that Glu 56 and Arg 60 of PfMLH interact with Glu 1386 and Glu 1387, which are present in the helicase C-terminal domain of PfUvrD (**Figures 6E,F**). We have previously reported that PfUvrD and PfMLH interact and modulate each other’s activities (Ahmad et al., 2012).

## Discussion

**FIGURE 7:**
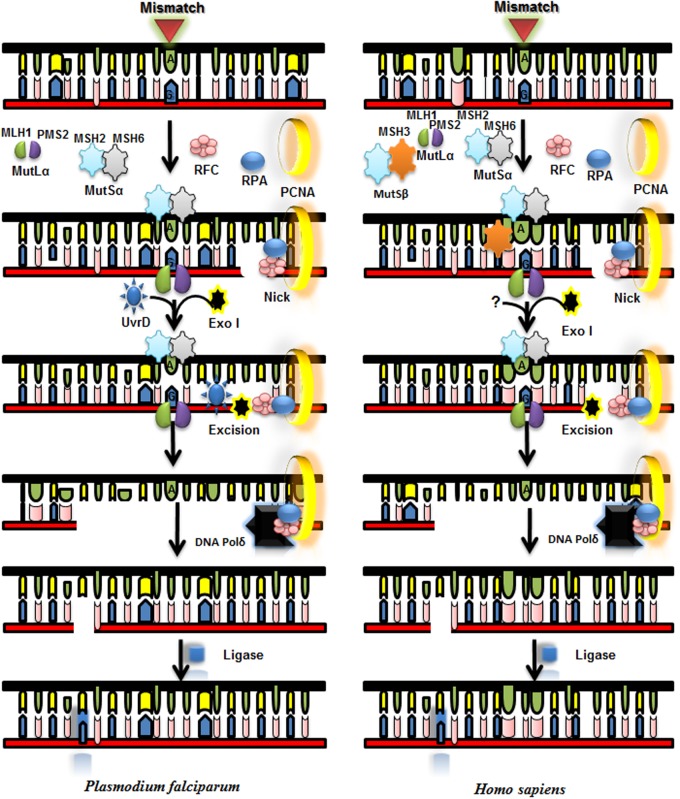
**Proposed model for mismatch repair (MMR) pathway of *P. falciparum* and its comparison with *Homo sapiens* MMR pathway**. MLH and MutS Homolog (MSH) complex and other interacting partners recognize the mismatch and MLH with its nuclease activity nicks near mismatched base. UvrD is recruited with the help of MLH and it unwinds the duplex DNA and Exo 1 degrades the unwound part of the duplex and then DNA polymerase synthesizes new DNA stretch and ligase seals the nick.

The key role of MMR pathway is to correct unpaired and mismatched bases in DNA as a result of erroneous replication and challenges faced by genome of an organism due to spontaneous and induced DNA modifications or due to recombination of genome. MMR is conserved from the single celled bacteria to multicellular humans. It involves three major steps which include recognition of mismatch; excision; and synthesis of excised DNA strand (Hsieh and Yamane, 2008). In humans, major components of MMR pathway are MutS and MutL, Exo1 (Exonuclease I), RPA (Replication Protein A), DNA polymerase δ, RFC (Replication Factor C), PCNA (proliferating cell nuclear antigen), and HMGB1 (Ahmad and Tuteja, 2014; Li, 2008). MMR pathway in *P. falciparum* is not well characterized and unprecedented as compared to humans. In *P. falciparum* MMR pathway two different protein complexes are involved for repair of 5′ nicked DNA; MutSα which includes MSH2-MSH6 heterodimer, ExoI, RPA, RFC, PCNA, and DNA polymerase δ. On the other hand for repair of 3′ nicked DNA in addition to all of these mentioned protein complexes MutLα (MLH1-PMS2) also takes part in the repair process (Castellini et al., 2011). Our previous reports show that PfUvrD interacts with PfMLH (Ahmad et al., 2012; Ahmad and Tuteja, 2014). *P. falciparum* lacks MSH3, MSH4, and MSH5 which are present in human but it contains UvrD that is absent in human. Although further work is required for establishing the role of MutL and UvrD in MMR pathway in *P. falciparum*. The roles of the MMR components of *P. falciparum* are purely speculative and further work will help in elucidation of this pathway (**Figure 7**; Supplementary Table S1).

## Conclusion

Human malaria is a major cause of morbidity worldwide particularly in tropical and subtropical regions (Tuteja, 2007). The emergence of drug resistance in the malaria parasite has potential to spread across the world (Dondorp et al., 2010; Amaratunga et al., 2012; Miotto et al., 2013) therefore it is necessary to unravel the mechanism and underlying causes of the development of drug resistance. Drug resistant malaria parasites have defective MMR so to obtain an insight of underlying causes it is the need of the hour to study the components of MMR complex of malaria parasite and compare them with the host. Various non-synonymous single nucleotide polymorphisms (SNP) have been located in genes involved in MMR pathways particularly MLH and UvrD (Fidock, 2013; Miotto et al., 2013). MMR protein, PfMLH is an endonuclease and is essential to restore the MMR in drug resistant strain of *P. falciparum*. The *in silico* characterization of the various components of the Plasmodium MMR pathway such as MLH, Pms1, MSH2-1, MSH2-2, and MSH6 components has revealed many characteristic features. The sequence analysis using *in silico* approaches revealed that Pms1, MSH2-1, MSH2-2, and MSH6 contain their characteristics motifs (**Figures 1**–**6A,B**). PfMLH possesses ∼52 % identity to the human MLH1 (Shankar and Tuteja, 2008) and PfPms1 showed ∼30% identity with human Pms1. PfMSH2-1 and PfMSH2-2 showed ∼34% and ∼33% identity with the human MSH2-1 and MSH2-2, respectively. Human contains two isoforms of MSH2 but *P. falciparum* has two MSH2 genes, which are located on different chromosomes showcasing an ancient duplication event rather than that of recent duplication. Presence of two PfMSH2 homologs is unusual as they are not being observed in other organisms. It is noteworthy that other apicomplexans possess only one MSH2 gene as opposed to two MSH2 genes present in Plasmodium species. It might be possible that PfMSH2-1 and PfMSH2-2 play role in other biological processes in addition to DNA repair. It is noteworthy that homologs to human MSH3, MSH4 and MSH5 were not detectable in *P. falciparum* 3D7 genome.

PfMSH6 showed ∼36% identity with human MSH6. The phylogenetic analysis revealed that PfMLH is closer to the yeast MLH1. Similarly, the phylogenetic analysis of PfPms1 revealed its closeness to yeast/human Pms1 protein and PfMSH2-1, PfMSH2-2, and PfMSH6 showed closeness to their counterpart in human/yeast. The structural modeling of Pms1N revealed its similarity to the N-terminal domain of yeast Pms1. While Pms1C terminal region showed similarity to the C-terminal domain of the *Saccharomyces cerevisiae* MutLα. The structural modeling of PfMSH2-1 and PfMSH2-2 exhibited their similarity to the human MSH2 protein. The modeling of PfMSH6 showed its similarity to MutSα protein of human. The structural analysis of PfPms1, PfMSH2-1, PfMSH2-2, and PfMSH6 suggests that parasite contains similar proteins as human and yeast but the absence of MutH homologs raised the possibility of its closeness to the MutH less bacterium. Interestingly the predicted structure of PfMLH, PfPms1, PfMSH2-1, PfMSH2-2, PfMSH6 and PfUvrD are steriochemically validated, so they can be utilized for the drug designing purpose.

To understand the MMR mechanism in detail, the interacting partners of the major MMR components have been also determined. Previously we have identified through *in-vitro* studies that PfMLH interacts with the PfUvrD helicase and this interaction is required to regulate their biochemical activities. In this report, the *in silico* identification of interacting partners of major MMR components of *P. falciparum* revealed several Plasmodium specific proteins in addition to the expected interacting partners (Supplementary Table S2). Overall this study provides a platform for detailed characterization of the MMR pathway and its components. We hope that this study will greatly encourage the researchers to explore the MMR pathway of the parasite in detail and will contribute significantly to understand the malaria parasite MMR pathway and its importance in drug resistance.

## Author Contributions

MT: Did all the alignments and wrote the results. MA: Interpreted the results and helped in writing. MC: Made the figures and did the String analysis. RT: Provided the concept of the study and wrote and corrected the complete manuscript including figures.

## Conflict of Interest Statement

The authors declare that the research was conducted in the absence of any commercial or financial relationships that could be construed as a potential conflict of interest.

## Footnotes

1 http://www.ncbi.nlm.nih.gov

2 http://plasmodb.org/plasmo/

3 http://www.ebi.ac.uk/Tools/msa/clustalw2/

4 http://www.ebi.ac.uk/Tools/msa/clustalo/

5 http://www.cgl.ucsf.edu/chimera

6 https://www.ebi.ac.uk/pdbsum/

7 http://bioinfo3d.cs.tau.ac.il

8 http://swissmodel.expasy.org/

9 http://swissmodel.expasy.org/
