# Supplementary material for: Genome Wide In silico Analysis of the Mismatch Repair Components of Plasmodium falciparum and Their Comparison with Human Host
Source: Front Microbiol. 2017 Feb 9;8:130. doi: 10.3389/fmicb.2017.00130 (PMC5298969; doi:10.3389/fmicb.2017.00130)
Supplement: Supplementary file 1 [file Table_1.DOCX]

**Supplementary Table 1: Comparative analysis of MMR components of *Homo sapiens* and *Plasmodium falciparum* 3D7**

| ***S.No.*** | ***Homo sapiens*** | ***Plasmodium falciparum* 3D7** | **Plasmodb ID** | | | **% Identity** |
| --- | --- | --- | --- | --- | --- | --- |
|  |  |  | **Old** | **New** | **Size** |  |
|  | **MLH1** | DNA mismatch repair protein MLH (MLH1) | PF11_0184 | PF3D7_1117800 | 1016 aa | 52 |
|  | **PMS1** | Mismatch repair protein pms1 homologue, putative | MAL7P1.145 | PF3D7_0726300 | 1330 aa | 30 |
|  | **MSH2-1** | DNA mismatch repair protein MSH2-1 | PF14_0254 | PF3D7_1427500 | 811 aa | 34 |
|  | **MSH2-2** | DNA mismatch repair protein MSH2-2 | MAL7P1.206 | PF3D7_0706700 | 873 aa | 33 |
|  | **MSH6** | DNA mismatch repair protein MSH6 | PFE0270c | PF3D7_0505500 | 1350 aa | 36 |
|  | **NA** | UvrD | PFE0705c | PF3D7_0514100 | 1441 aa | NA |
