# Supplementary material for: Genome Wide In silico Analysis of the Mismatch Repair Components of Plasmodium falciparum and Their Comparison with Human Host
Source: Front Microbiol. 2017 Feb 9;8:130. doi: 10.3389/fmicb.2017.00130 (PMC5298969; doi:10.3389/fmicb.2017.00130)
Supplement: Supplementary file 2 [file Table_2.DOCX]

**Supplementary Table 2: Interacting Partners of MMR components of *Plasmodium falciparum* 3D7**

| **S.no.** | **Name and PlasmoDB no.**  **(old/new)** | **PlasmoDB no. old/new of interacting partners** | **Name of interacting partners**  **(size in amino acids)** | **Score** |
| --- | --- | --- | --- | --- |
| **_1._** | **_MLH_**  **_PF11_0184/ PF3D7_1117800_** | 1. _PF14_0254/PF3D7_1427500_ 2. _PFE0270c/PF3D7_0505500_ 3. _PFE0705c/PF3D7_0514100_ 4. _MAL7P1.206/PF3D7_0706700_ 5. _PF07_0105/PF3D7_0725000_ 6. _PFI0910w/PF3D7_0918600_ 7. _PF14_0278/PF3D7_1429900_ 8. _PF13_0328/PF3D7_1361900_ 9. _PF3D7_1226600)_ 10. _MAL7P1.145/PF3D7_0726300_ | _DNA mismatch repair enzyme (811 aa)_  _DNA repair protein, putative (1350 aa)_  _Helicase, belonging to UvrD family, putative (1441 aa)_  _DNA mismatch repair protein, putative (873 aa)_  _Exonuclease I, putative (1347 aa)_  _DNA helicase, putative (728 aa)_  _ATP-dependent DNA helicase, putative (1440 aa)_  _Proliferating cell nuclear antigen 1 (274 aa)_  _Proliferating cell nuclear antigen 2 (264 aa_  _MMR protein pms1 homologue, putative (1330 aa)_ | _0.998_  _0.998_  _0.996_  _0.996_  _0.994_  _0.979_  _0.978_  _0.975_  _0.972_  _0.968_ |
| **_2._** | **_Pms1_**  **_MAL7P1.145/ PF3D7_0726300_** | 1. _PF14_0254/PF3D7_1427500_ 2. _MAL7P1.206/PF3D7_0706700_ 3. _PFE0270c/PF3D7_0505500_ 4. _PF11_0184/PF3D7_1117800_ 5. _PF07_0105/PF3D7_0725000_ 6. _PFE0705c/PF3D7_0514100_ 7. _PFL0380c/PF3D7_1207600)_ | _DNA mismatch repair enzyme (811 aa)_  _DNA mismatch repair protein, putative (873 aa)_  _DNA repair protein, putative (1350 aa)_  _DNA mismatch repair enzyme (1016 aa)_  _Exonuclease I, putative (1347 aa)_  _Helicase, belonging to UvrD family, putative (1441 aa)_  _tRNA delta(2)-isopentenylpyrophosphate transferase, putative(601 aa)_ | _0.996_  _0.996_  _0.995_  _0.968_  _0.967_  _0.935_  _0.908_ |
| **_3._** | **_MSH2-1_**  **_PF14_0254/ PF3D7_1427500_** | 1. _PF11_0184/PF3D7_1117800)_ 2. _MAL7P1.145/PF3D7_0726300_ 3. _PF13_0328/PF3D7_1361900_ 4. _PF07_0105/PF3D7_0725000_ 5. _PF3D7_1226600_ 6. _PFI0910w/PF3D7_0918600_ 7. _PFB0160w/PF3D7_0203300_ 8. _PFF1470c/PF3D7_0630300_ 9. _PFI1650w/PF3D7_0934100_ 10. _PF14_0278/PF3D7_1429900_ | _DNA mismatch repair enzyme (1016 aa)_  _MMR protein pms1 homologue, putative (1330 aa)_  _Proliferating cell nuclear antigen 1 (274 aa)_  _Exonuclease I, putative (1347 aa)_  _Proliferating cell nuclear antigen 2 (264 aa)_  _DNA helicase, putative (728 aa)_  _ERCC1 NER protein, putative (242 aa)_  _DNA polymerase epsilon, subunit a, putative (2907 aa)_  _DNA excision-repair helicase, putative (1056 aa)_  _ATP-dependent DNA helicase, putative (1440 aa)_ | _0.998_  _0.996_  _0.986_  _0.983_  _0.979_  _0.972_  _0.968_  _0.966_  _0.961_  _0.961_ |
| **_4._** | **_MSH2-2_**  **_MAL7P1.206/ PF3D7_0706700_** | 1. _PF11_0184/PF3D7_1117800_ 2. _MAL7P1.145/PF3D7_0726300_ 3. _PF07_0105/PF3D7_0725000_ 4. _PF13_0328/PF3D7_136190_ 5. _PF3D7_1226600)_ 6. _PFB0160w/PF3D7_0203300_ 7. _PFI0910w/PF3D7_0918600)_ 8. _PF14_0278/PF3D7_1429900_ 9. _PF3D7_1107400_ 10. _MAL8P1.65/PF3D7_0818700_ | _DNA mismatch repair enzyme (1016 aa)_  _MMR protein pms1 homologue, putative (1330 aa)_  _Exonuclease I, putative (1347 aa)_  _Proliferating cell nuclear antigen1 (274 aa)_  _Proliferating cell nuclear antigen 2 (264 aa)_  _ERCC1 NER repair protein, putative (242 aa)_  _DNA helicase, putative (728 aa)_  _ATP-dependent DNA helicase, putative (1440 aa)_  _Recombinase Rad51 (350 aa)_  _DNA helicase, putative (1221 aa)_ | _0.996_  _0.996_  _0.979_  _0.977_  _0.974_  _0.970_  _0.956_  _0.956_  _0.932_  _0.926_ |
| **_5._** | **_MSH6_**  **_PFE0270c/ PF3D7_0505500_** | 1. _PF11_0184/PF3D7_1117800_ 2. _MAL7P1.145/PF3D7_072630_ 3. _PF13_0328/PF3D7_1361900_ 4. _PF3D7_1226600_ 5. _PFF1470c/PF3D7_0630300_ 6. _PF14_0254/PF3D7_1427500_ 7. _PF3D7_1107400_ 8. _PFI0235w/PF3D7_0904800_ 9. _PFI0910w/PF3D7_0918600_ 10. _PF13_0095/PF3D7_1317100_ | _DNA mismatch repair enzyme (1016 aa)_  _MMR protein pms1 homologue, putative (1330 aa)_  _Proliferating cell nuclear antigen 1 (274 aa)_  _Proliferating cell nuclear antigen 2 (264 aa)_  _DNA polymerase epsilon, subunit a, putative (2907 aa)_  _DNA mismatch repair enzyme (811 aa)_  _Recombinase Rad51 (350 aa)_  _Replication factor A-related protein, putative (484 aa)_  _DNA helicase, putative (728 aa)_  _DNA replication licensing factor MCM4-related (1005 aa)_ | _0.998_  _0.995_  _0.995_  _0.986_  _0.968_  _0.945_  _0.933_  _0.932_  _0.924_  _0.915_ |
| **_6._** | **_UvrD_**  **_PFE0705c/ PF3D7_0514100_** | 1. _F11_0184/PF3D7_1117800_ 2. _MAL7P1.145/PF3D7_0726300_ | _DNA mismatch repair enzyme (1016 aa)_  _MMR protein pms1 homologue, putative (1330 aa)_ | _0.996_  _0.935_ |
