# Supplementary material for: Genome Wide In silico Analysis of the Mismatch Repair Components of Plasmodium falciparum and Their Comparison with Human Host
Source: Front Microbiol. 2017 Feb 9;8:130. doi: 10.3389/fmicb.2017.00130 (PMC5298969; doi:10.3389/fmicb.2017.00130)
Supplement: Supplementary file 3 [file Presentation_1.PDF]

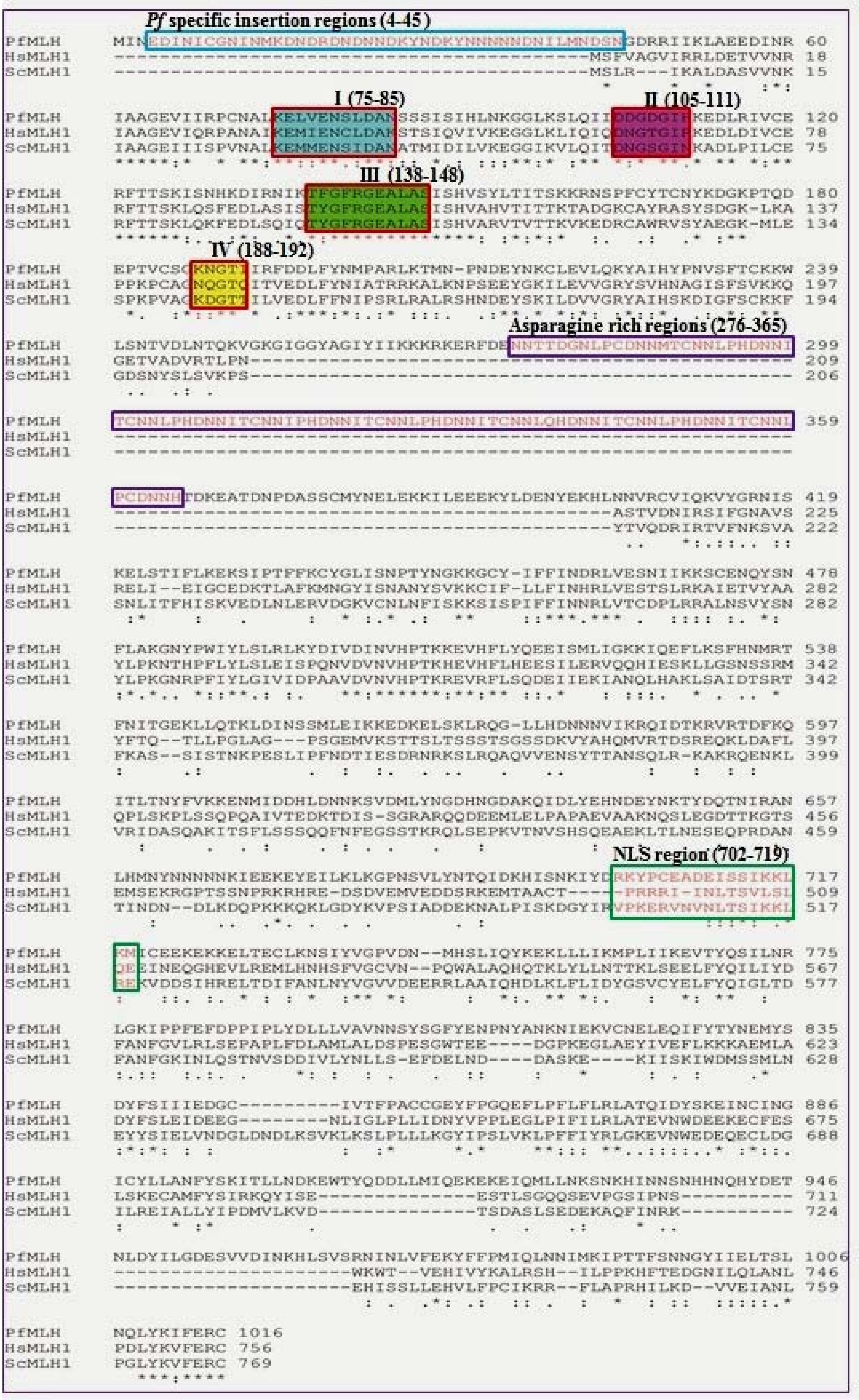

Pf specific insertion regions (4-45)
I (75-85)
II (105-111)
III (138-148)
IV (188-192)
Asparagine rich regions (276-365)
NLS region (702-719)
PfMLH
HsMLH1
ScMLH1

S1-A

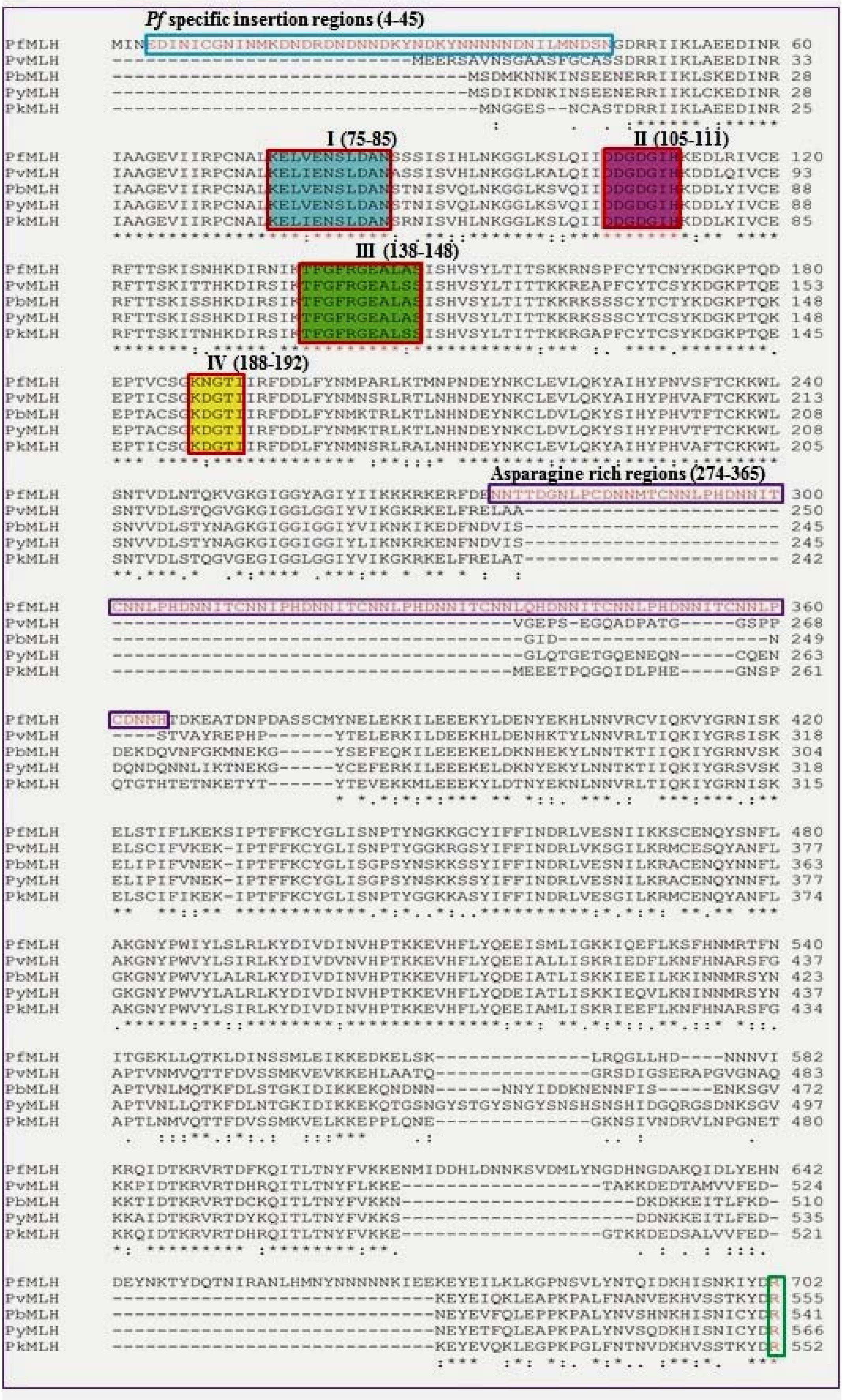

Pf specific insertion regions (4-45)
I (75-85)
II (105-111)
III (138-148)
IV (188-192)
Asparagine rich regions (274-365)
PfMLH
PvMLH
PbMLH
PyMLH
PkMLH

S1-B

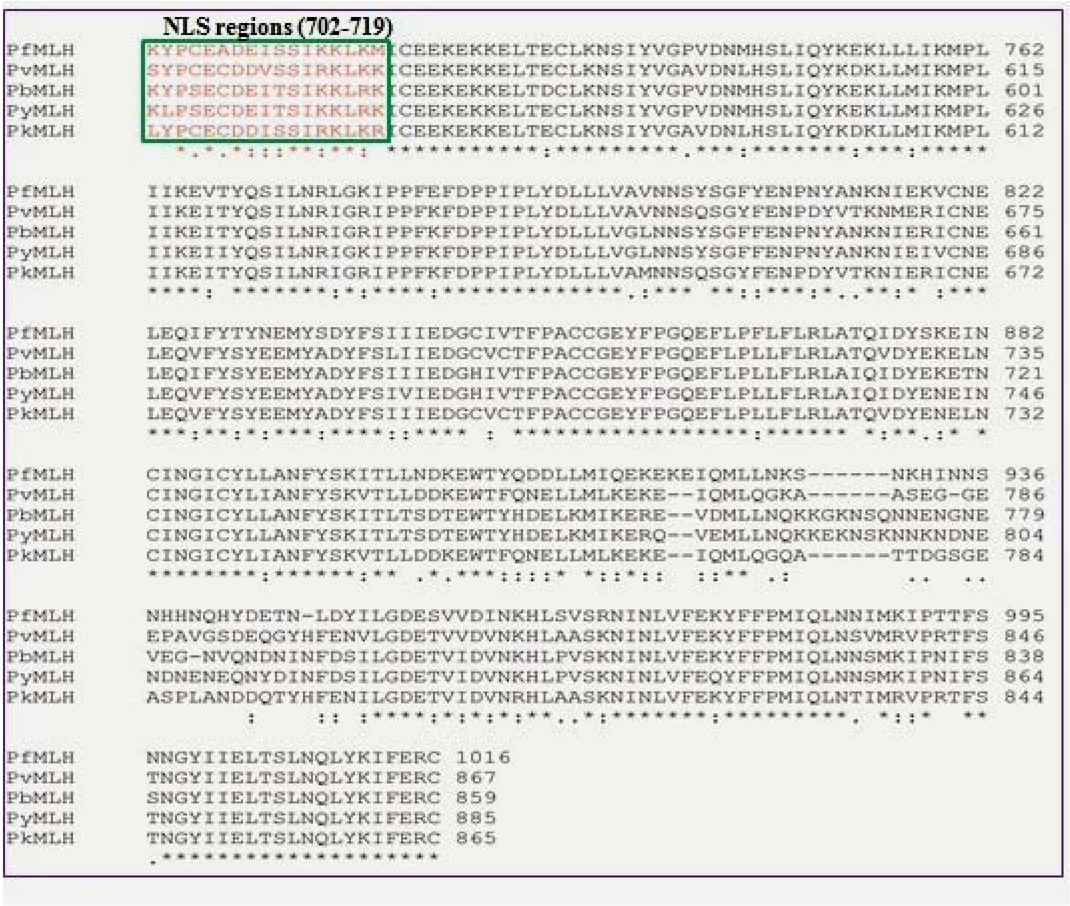
NLS regions (702-719)
PfMLH KYPCEADEISSIKKLKM ICEEKEKKELTECLKNSIYVGPVDNMHSLIQYKEKLLLIKMPL 762
PvMLH SYPCECDDVSSIRKLKK ICEEKEKKELTECLKNSIYVGAVDNLHSLIQYKDKLLMIKMPL 615
PbMLH KYPSECDEITSIKKLRK ICEEKEKKELTDCLKNSIYVGPVDNMHSLIQYKEKLLMIKMPL 601
PyMLH KLPSECDEITSIKKLRK ICEEKEKKELTECLKNSIYVGPVDNMHSLIQYKEKLLMIKMPL 626
PkMLH LYPCECDDISSIRKLKR ICEEKEKKELTECLKNSIYVGAVDNLHSLIQYKDKLLMIKMPL 612
PfMLH IIKEVTYQSILNRLGKIPPFEFDPPIPLYDLLLVAVNNSYSGFYENPNYANKNIEKVCNE 822
PvMLH IIKEITYQSILNRIGRIPPFKFDPPIPLYDLLLVAVNNSQSGYFENPDYVTKNMERICNE 675
PbMLH IIKEITYQSILNRIGRIPPFKFDPPIPLYDLLLVGLNNSYSGFFENPNYANKNIERICNE 661
PyMLH IIKEIIYQSILNRIGKIPPFKFDPPIPLYDLLLVGLNNSYSGFFENPNYANKNIEIVCNE 686
PkMLH IIKEITYQSILNRIGRIPPFKFDPPIPLYDLLLVAMNNSQSGYFENPDYVTKNIERICNE 672
PfMLH LEQIFYTYNEMYSDYFSIIIEDGCIVTFPACCGEYFPGQEFLPFLFLRLATQIDYSKEIN 882
PvMLH LEQVFYSYEEMYADYFSLIIEDGCVCTFPACCGEYFPGQEFLPLLFLRLATQVDYEKELN 735
PbMLH LEQIFYSYEEMYADYFSIIIEDGHIVTFPACCGEYFPGQEFLPLLFLRLAIQIDYEKETN 721
PyMLH LEQVFYSYEEMYADYFSIVIEDGHIVTFPACCGEYFPGQEFLPLLFLRLAIQIDYENEIN 746
PkMLH LEQVFYSYEEMYADYFSIIIEDGCVCTFPACCGEYFPGQEFLPLLFLRLATQVDYENELN 732
PfMLH CINGICYLLANFYSKITLLNDKEWTYQDDLLMIQEKEKEIQMLLNKS------NKHINNS 936
PvMLH CINGICYLIANFYSKVTLLDDKEWTFQNELLMLKEKE--IQMLQGKA------ASEG-GE 786
PbMLH CINGICYLLANFYSKITLTSDTEWTYHDELKMIKERE--VDMLLNQKKGKNSQNNENGNE 779
PyMLH CINGICYLLANFYSKITLTSDTEWTYHDELKMIKERQ--VEMLLNQKKEKNSKNNKNDNE 804
PkMLH CINGICYLIANFYSKVTLLDDKEWTFQNELLMLKEKE--IQMLQGQA------TTDGSGE 784
PfMLH NHHNQHYDETN-LDYILGDESVVDINKHLSVSRNINLVFEKYFFPMIQLNNIMKIPTTFS 995
PvMLH EPAVGSDEQGYHFENVLGDETVVDVNKHLAASKNINLVFEKYFFPMIQLNSVMRVPRTFS 846
PbMLH VEG-NVQNDNINFDSILGDETVIDVNKHLPVSKNINLVFEKYFFPMIQLNNSMKIPNIFS 838
PyMLH NDNENEQNYDINFDSILGDETVIDVNKHLPVSKNINLVFEQYFFPMIQLNNSMKIPNIFS 864
PkMLH ASPLANDDQTYHFENILGDETVIDVNRHLAASKNINLVFEKYFFPMIQLNTIMRVPRTFS 844
PfMLH NNGYIIELTSLNQLYKIFERC 1016
PvMLH TNGYIIELTSLNQLYKIFERC 867
PbMLH SNGYIIELTSLNQLYKIFERC 859
PyMLH TNGYIIELTSLNQLYKIFERC 885
PkMLH TNGYIIELTSLNQLYKIFERC 865

S1-B

```
                                                         ATPase domain (1-218)
PfPms1  --------------------------------MKIKNIGEESIHNICSSQVIFTLSSVVK 28
ScPms1  MFHHIENLLIETEKRCKQKEQRYIPVKYLFSMTQIHQINDIDVHRITSGQVITDLTTAVK 60
HsPms2  ---------MERA-----------ESSSTEPAKAIKPIDRKSVHQICSGQVVLSLSTAVK 40
                                          *: *.  .:*.* *.**:  *::.**

PfPms1  ELVENSIDADASEIKIKLVESGIKLIEVNDNGVGIKKINFENICARHATSKIKDFNDIHS 88
ScPms1  ELVDNSIDANANQIEIIFKDYGLESIECSDNGDGIDPSNYEFLALKHYTSKIAKFQDVA- 119
HsPms2  ELVENSLDAGATNIDLKLKDYGVDLIEVSDNGCGVEEENFEGLTLKHHTSKIQEFADLT- 99
        ***:**:**.*.:*.: : : *:. ** .*** *:.  *:* :  :* **** .* *:

PfPms1  SLNTLGFRGEALNSLCMLSNVNITTKNEENDHAYLLKFDKLGRLYHEEPIARLRGTTVSC 148
ScPms1  KVQTLGFRGEALSSLCGIAKLSVITTTSP-PKADKLEYDMVGHITSKTTTSRNKGTTVLV 178
HsPms2  QVETFGFRGEALSSLCALSDVTISTCHASAKVGTRLMFDHNGKIIQKTPYPRPRGTTVSV 159
        .::*:*******.*** ::.:.: *          .  * :*  *::  :    * :****

PfPms1  ENIFHNIPIRKKDFIKNIKTQVSDLLLLMQQYAIIYHNIKFVIYNIVTSKGCTKKMNLLI 208
ScPms1  SQLFHNLPVRQKEFSKTFKRQFTKCLTVIQGYAIINAAIKFSVWNITPK---GKKNLILS 235
HsPms2  QQLFSTLPVRHKEFQRNIKKEYAKMVQVLHAYCIISAGIRVSCTNQLGQ---GKRQPVVC 216
        .::* .:*:*:*:* :.:* : :. : :: *.** *.**   *:.    *    .    *.  ::
 MMR domain (202-236)
PfPms1  TNGTDSIKKNFYSIYGKRNIGNLIEFNID-----------------------GNEWNIRG 245
ScPms1  TMRNSSMRKNISSVFGAGGMRGLEEVDLVLDLNPFKNRMLGKYTDDPDFLDLDYKIRVKG 295
HsPms2  TGGSPSIKENIGSVFGQKQLQSLIPFV---QLPPSD-SVCEEYGLS-CSDALHNLFYISG 271
        *  . *:::*: *::*   : .*  .                                : *

PfPms1  YISDS--NSGRRDKDLQFYYINSRPIHILKNVNKLINTIYREFNSRLYPIIICNILSDTK 303
ScPms1  YISQNSFGCGRNSKDRQFIYVNKRPVEYS-TLLKCCNEVYKTFNNVQFPAVFLNLELPMS 354
HsPms2  FISQCTHGVGRSSTDRQFFFINRRPCDPA-KVCRLVNEVYHMYNRHQYPFVVLNISVDSE 330
        :**:    . ** ..* ** ::* ** .    .: :  * :*: :*    :* :. *:    .

PfPms1  NIDINVTPDKREVFFTFEQEMCEHMKTALVKLFTPKTSNLIDTQIDDYFLKANNILPNQI 363
ScPms1  LIDVNVTPDKRVILLHNERAVIDIFKTTLSDYYNRQELALPK------------------ 396
HsPms2  CVDINVTPDKRQILLQEEKLLLAVLKTSLIGMFDSDVNKLNVSQQPLLDVEGN------- 383
         :*:******* :::  *: :   :**:*   :  .   *

PfPms1  KSNIKLETHEHDDNHHHHKSTSPSQKHKGNEKPNDNIIKMEPNYNNEVTHHAYVKKELIH 423
ScPms1  -------------RM-CSQSEQQAQKRLK-----------TEVFDDRST----------- 420
HsPms2  --LIKMHAADLEKPM-VEKQDQSPSLRTGEEKKDVSISRLREAFSLRHT----------- 429
                         :. .  . :                  :. . *
                                                 DNA binding domain (486-490)
PfPms1  KDDQIYVKQEEMKDQNNSYHIEKDNDIDAEHCNRHEFGEEKENIFTYHDDRQLFKIKEEK 483
ScPms1  ----------THESDNENYHTARSESN---QSNHAHFNSTTGVI-DKSNGTELT------ 460
HsPms2  -------------TENKP-HSPK--TP---EPRRSPLGQKRGML-SSST----------- 458
                     :*:  *  :        . .: :..    :

PfPms1  IYNYPNKYIENDELYRDNTSSLSYTLSQKDYIPTENKMNNNIQIKNDELNSSSLFQNDDY 543
ScPms1  ------------------------------SVMDGNYTNVTD------------- 472
HsPms2  ---------------------------------SGAISDK-G------------- 466
                                         ..       .

PfPms1  NFSNIYRANHSFTSTQESMHCDLFVSGNKKQYENSEKEKNEKNVENKKNTEYTFFRNNEE 603
ScPms1  ----VI-----------GSECEVSVDSSVVLDEGNSSTP-------------------T 497
HsPms2  ----VL-----------RPQ------KEAVSSSHGPSDP-------------------T 485
            :            .         .     . . .

PfPms1  QKSGYIKKGGEEFNTYNDEEIYSSGPLSIDNVMMENNKEHFSDMNNNVYEYKLNNDDTKN 663
ScPms1  KKLPSIKTDSQNL---------------------------SDLNLNNFS----NPEFQN 525
HsPms2  DRAEVEKDSGHGS---------------------------TSVDSEGFS----IPDTGS 513
        .:     * ...                            :.:: : :.       :  .

PfPms1  -NIYEYKLNSDDTKNNIYEYKLNNDDTKNNIYEYKLNNDDTKNNIYEYKLNNDDSNNTIY 722
ScPms1  ITS-------PDKARSL-----------EKVVE---------EPVY-FDIDGEKFQEK-- 555
HsPms2  HCSSEYAASSPGDRGSQ-----------EHVD---------------------SQE--- 537
```

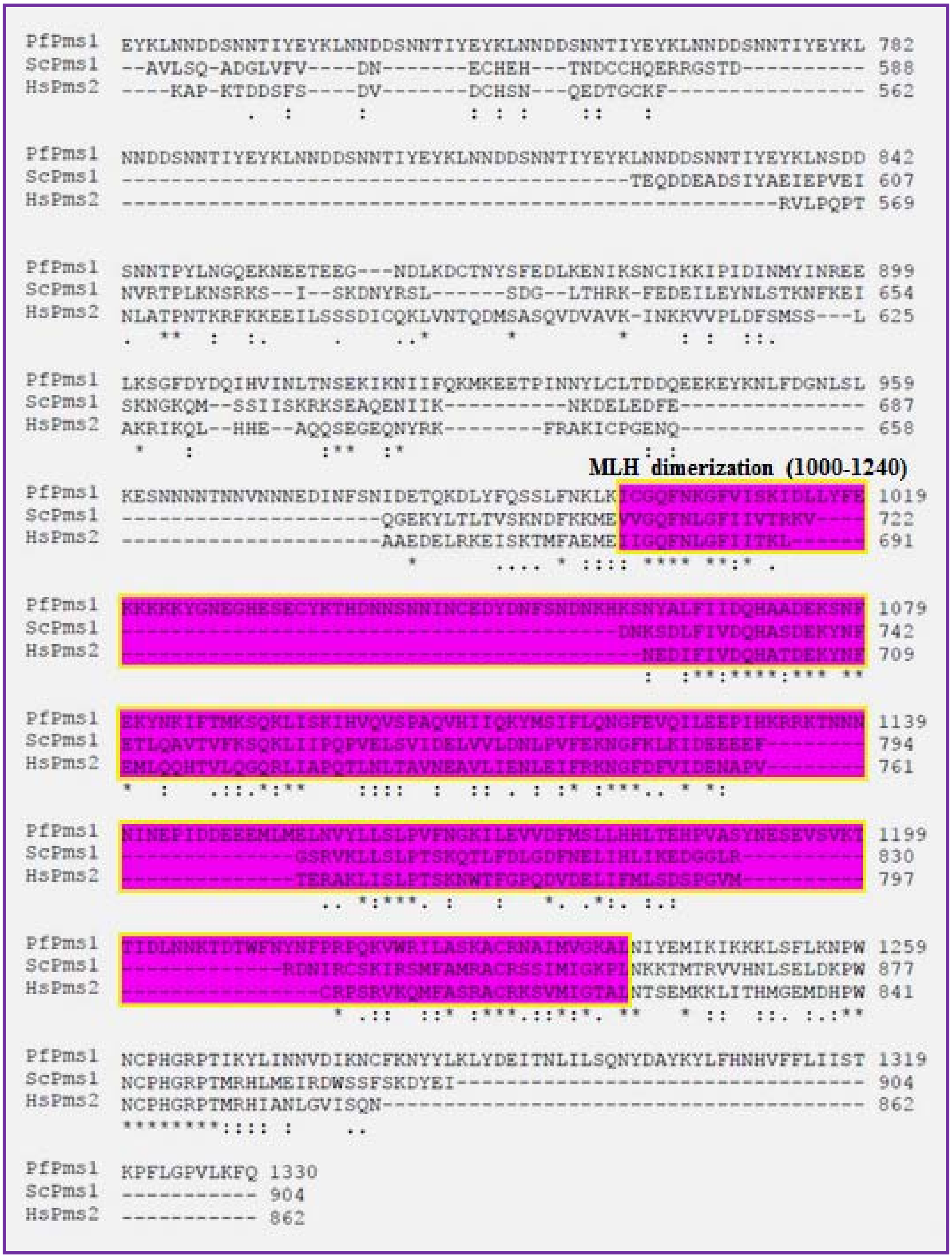

S2-A

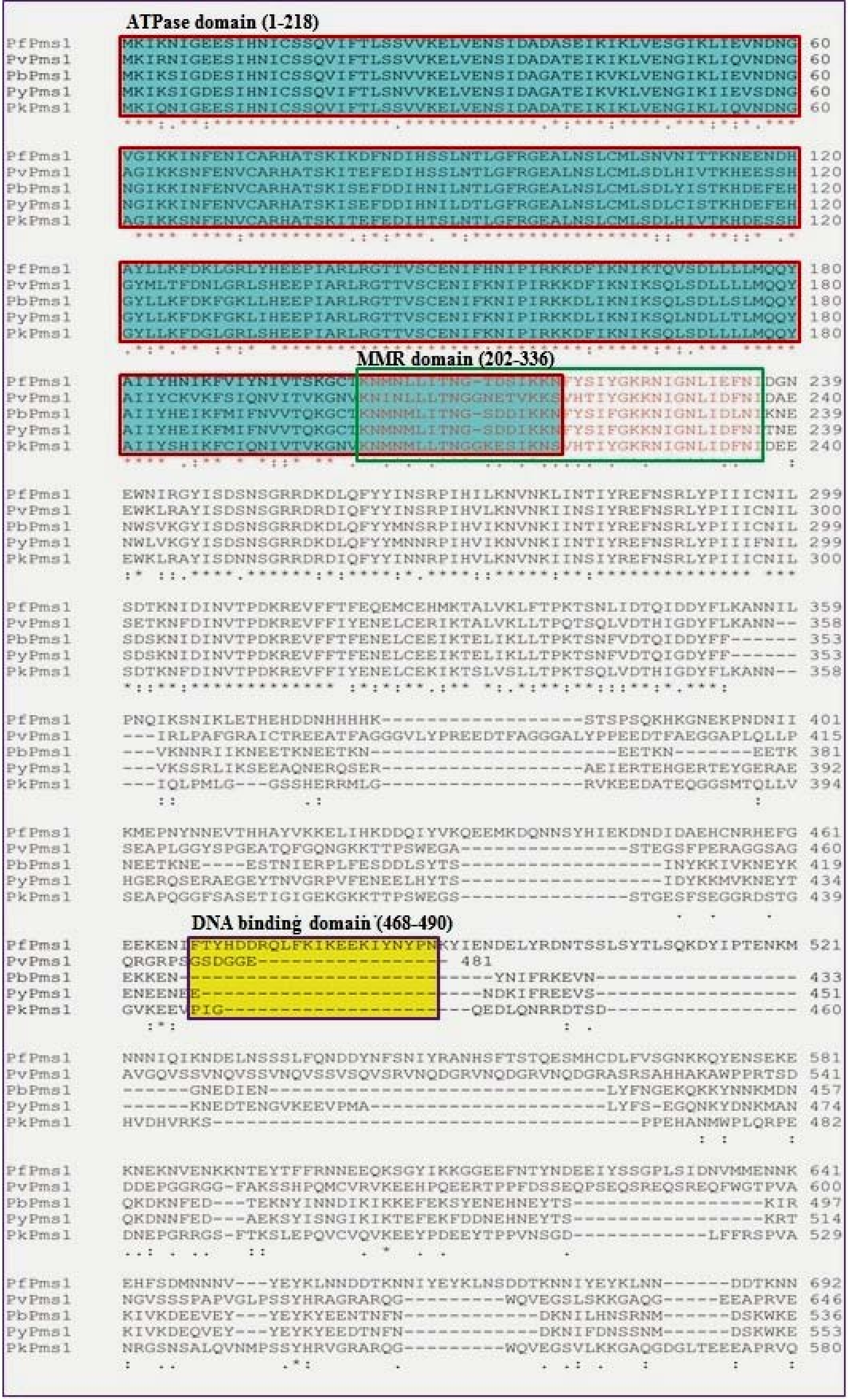
ATPase domain (1-218)
MMR domain (202-336)
DNA binding domain (468-490)
PfPms1
PvPms1
PbPms1
PyPms1
PkPms1

S2-B

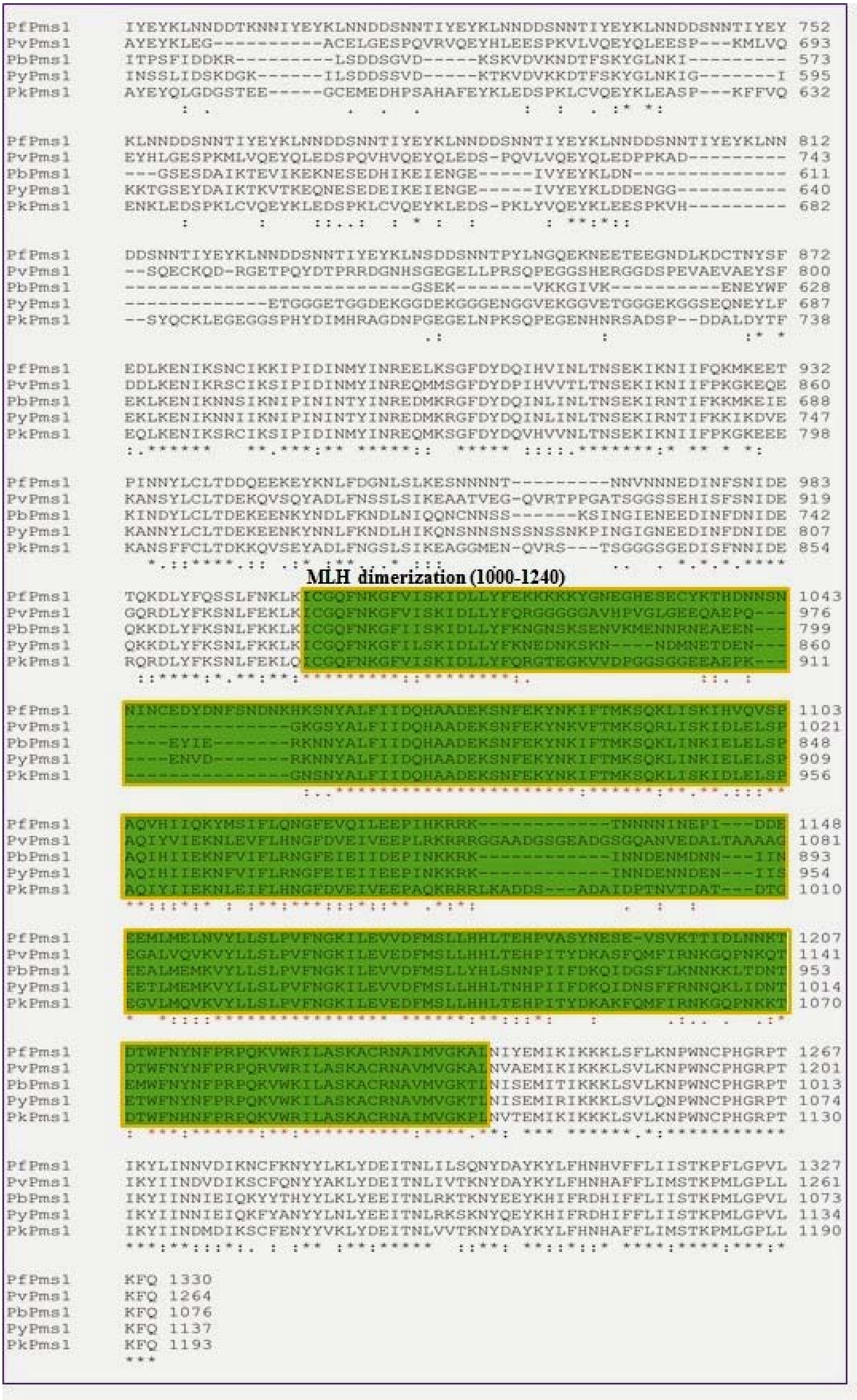

S2-B

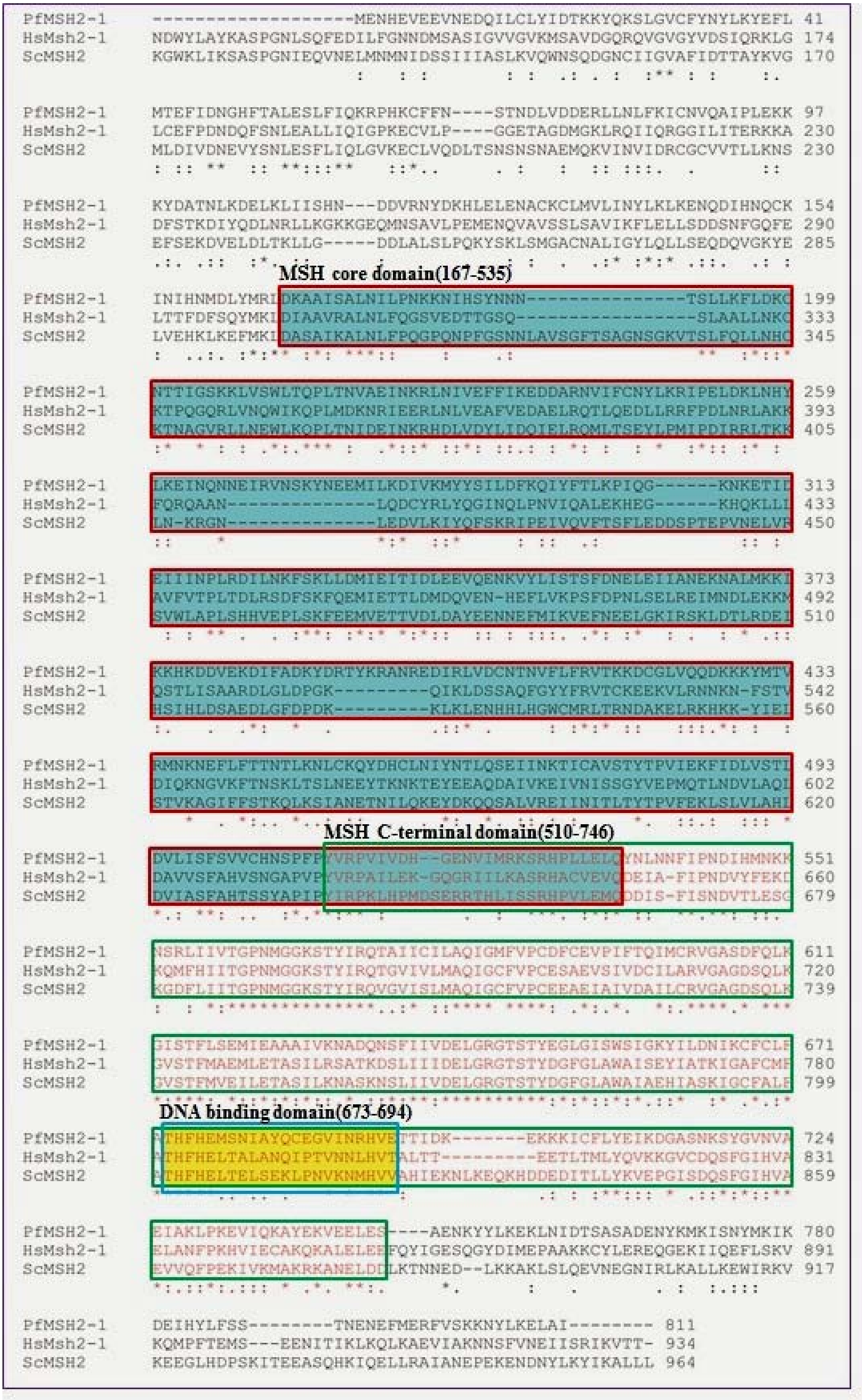
PfMSH2-1
HsMsh2-1
ScMSH2
MSH core domain(167-535)
MSH C-terminal domain(510-746)
DNA binding domain(673-694)

S3-A

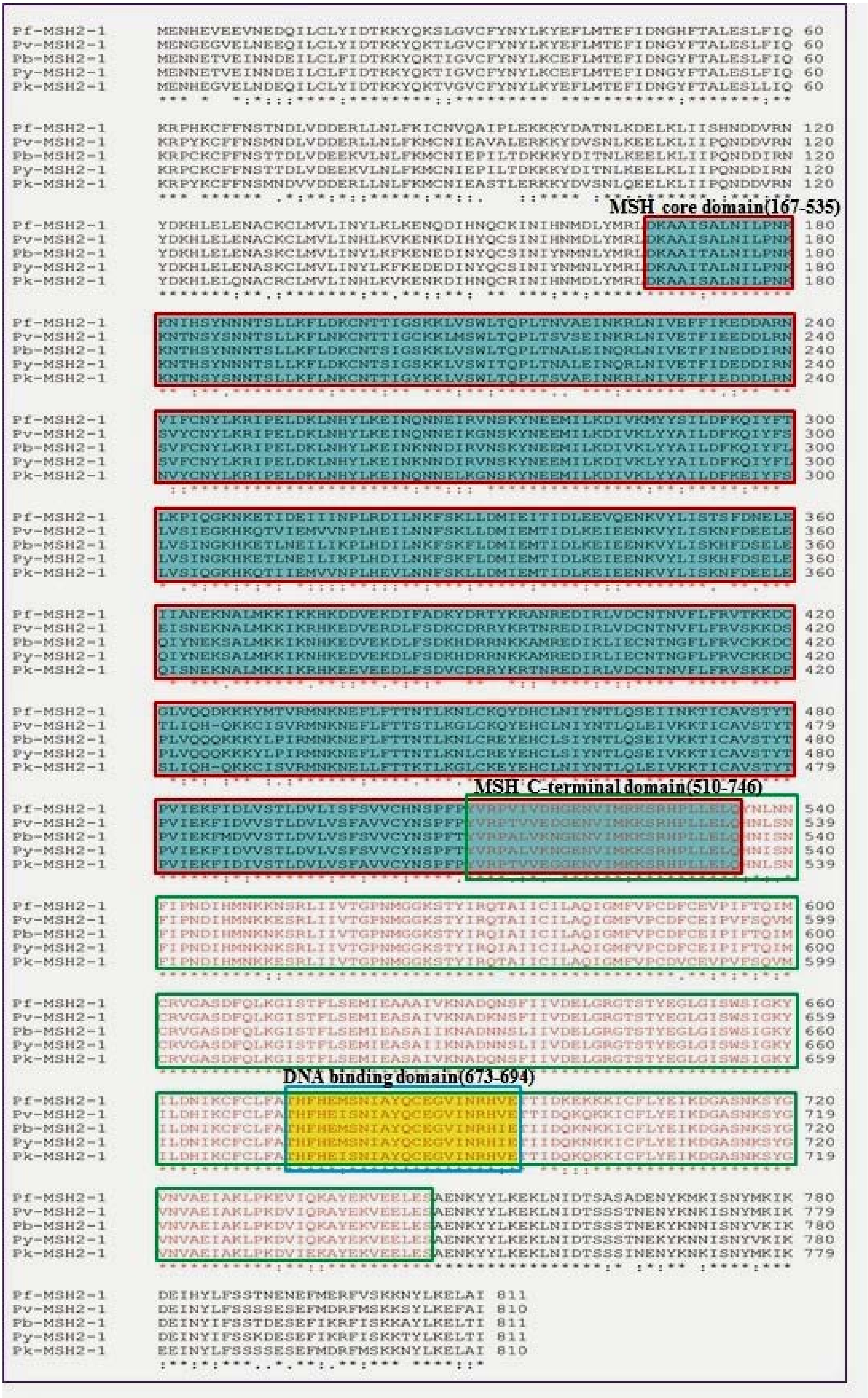
MSH core domain(167-535)
MSH C-terminal domain(510-746)
DNA binding domain(673-694)
Pf-MSH2-1
Pv-MSH2-1
Pb-MSH2-1
Py-MSH2-1
Pk-MSH2-1

S3-B

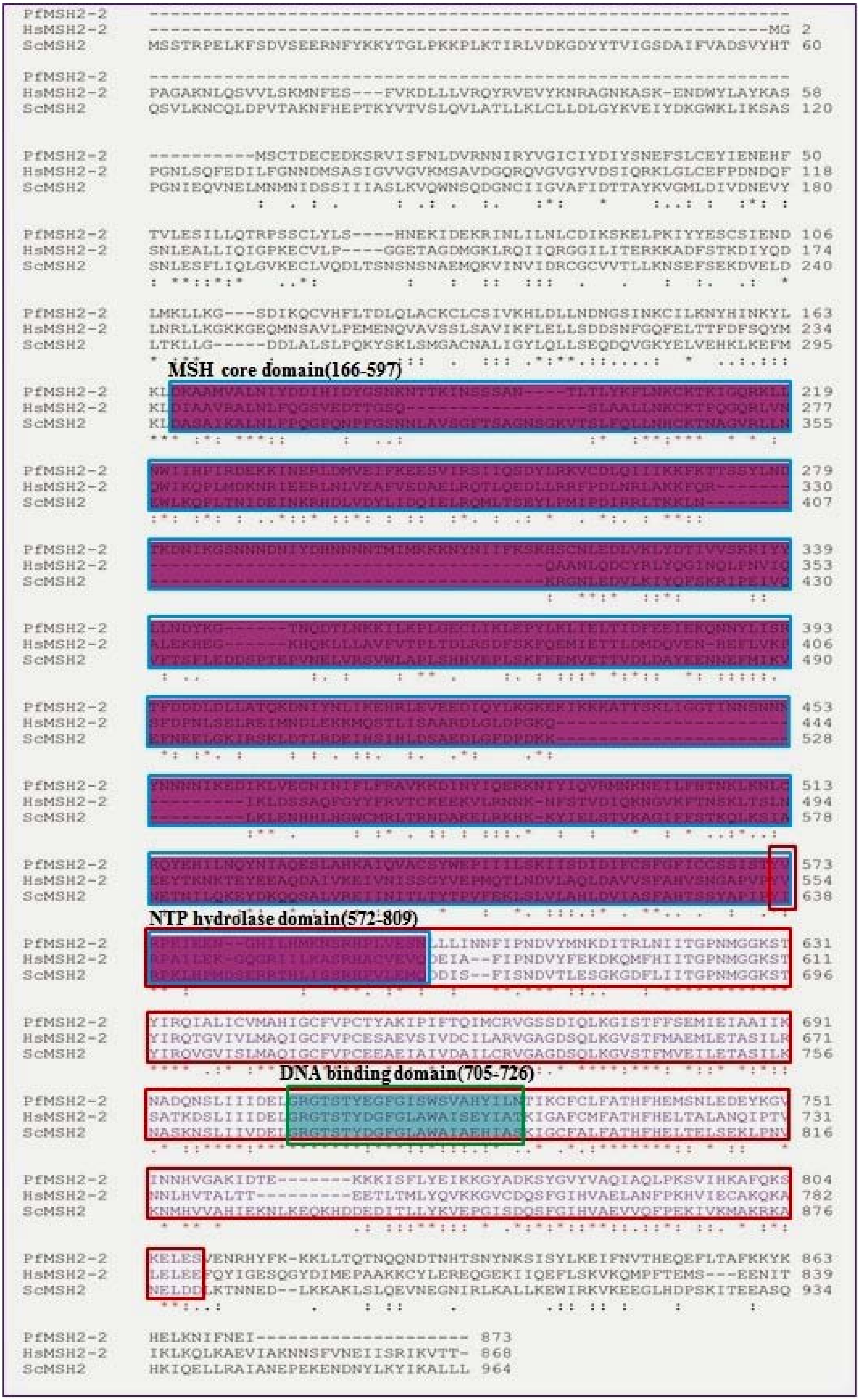
PfMSH2-2
HsMSH2-2
ScMSH2
MSH core domain(166-597)
NTP hydrolase domain(572-809)
DNA binding domain(705-726)

S4-A

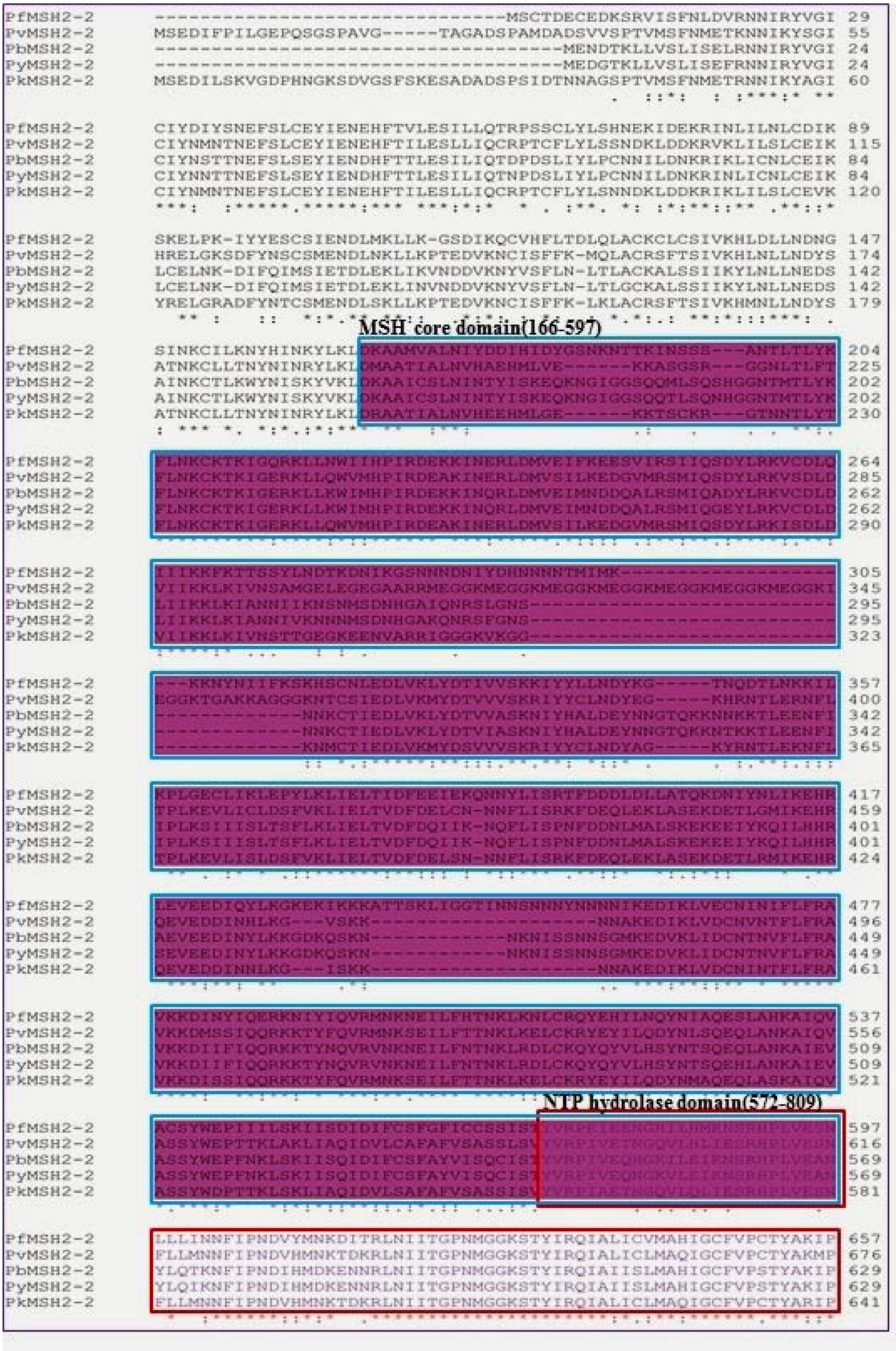

S4-B

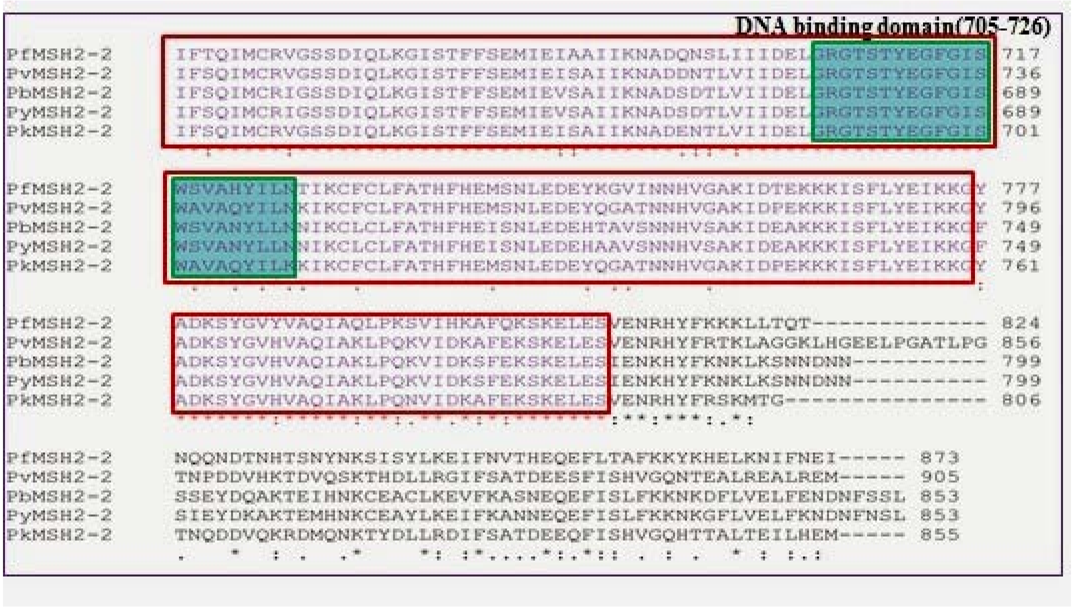
DNA binding domain(705-726)
PfMSH2-2 IFTQIMCRVGSSDIQLKGISTFFSEMIEIAAIIKNADQNSLIIIDELGRGTSTYEGFGIS 717
PvMSH2-2 IFSQIMCRVGSSDIQLKGISTFFSEMIEISAIIKNADDNTLVIIDELGRGTSTYEGFGIS 736
PbMSH2-2 IFSQIMCRIGSSDIQLKGISTFFSEMIEVSAIIKNADSDTLVIIDELGRGTSTYEGFGIS 689
PyMSH2-2 IFSQIMCRIGSSDIQLKGISTFFSEMIEVSAIIKNADSDTLVIIDELGRGTSTYEGFGIS 689
PkMSH2-2 IFSQIMCRVGSSDIQLKGISTFFSEMIEISAIIKNADENTLVIIDELGRGTSTYEGFGIS 701
PfMSH2-2 WSVAHYILNTIKCFCLFATHFHEMSNLEDEYKGVINNHVGAKIDTEKKKISFLYEIKKGY 777
PvMSH2-2 WAVAQYILNKIKCFCLFATHFHEMSNLEDEYQGATNNHVGAKIDPEKKKISFLYEIKKGY 796
PbMSH2-2 WSVANYLLNNIKCLCLFATHFHEISNLEDEHTAVSNNHVSAKIDEAKKKISFLYEIKKGF 749
PyMSH2-2 WSVANYLLNNIKCLCLFATHFHEISNLEDEHAAVSNNHVSAKIDEAKKKISFLYEIKKGF 749
PkMSH2-2 WAVAQYILKKIKCFCLFATHFHEMSNLEDEYQGATNNHVGAKIDPEKKKISFLYEIKKGY 761
PfMSH2-2 ADKSYGVYVAQIAQLPKSVIHKAFQKSKELESVENRHYFKKKLLTQT------------- 824
PvMSH2-2 ADKSYGVHVAQIAKLPQKVIDKAFEKSKELESVENRHYFRTKLAGGKLHGEELPGATLPG 856
PbMSH2-2 ADKSYGVHVAQIAKLPQKVIDKSFEKSKELESIENKHYFKNKLKSNNDNN---------- 799
PyMSH2-2 ADKSYGVHVAQIAKLPQKVIDKSFEKSKELESIENKHYFKNKLKSNNDNN---------- 799
PkMSH2-2 ADKSYGVHVAQIAKLPQNVIDKAFEKSKELESVENRHYFRSKMTG--------------- 806
PfMSH2-2 NQQNDTNHTSNYNKSISYLKEIFNVTHEQEFLTAFKKYKHELKNIFNEI----- 873
PvMSH2-2 TNPDDVHKTDVQSKTHDLLRGIFSATDEESFISHVGQNTEALREALREM----- 905
PbMSH2-2 SSEYDQAKTEIHNKCEACLKEVFKASNEQEFISLFKKNKDFLVELFENDNFSSL 853
PyMSH2-2 SIEYDKAKTEMHNKCEAYLKEIFKANNEQEFISLFKKNKGFLVELFKNDNFNSL 853
PkMSH2-2 TNQDDVQKRDMQNKTYDLLRDIFSATDEEQFISHVGQHTTALTEILHEM----- 855

S4-B

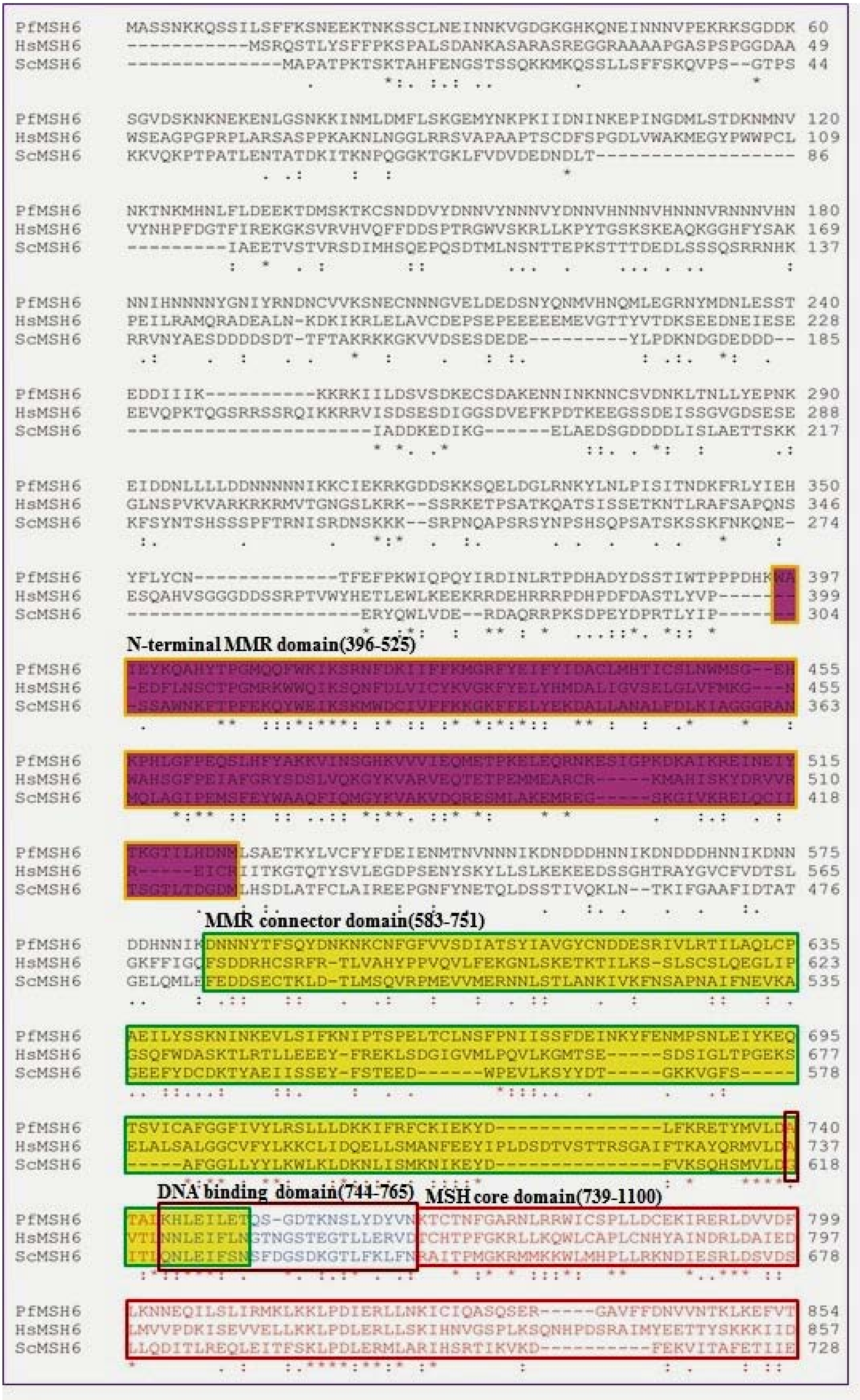
PfMSH6
HsMSH6
ScMSH6
N-terminal MMR domain(396-525)
MMR connector domain(583-751)
DNA binding domain(744-765)
MSH core domain(739-1100)

S5-A

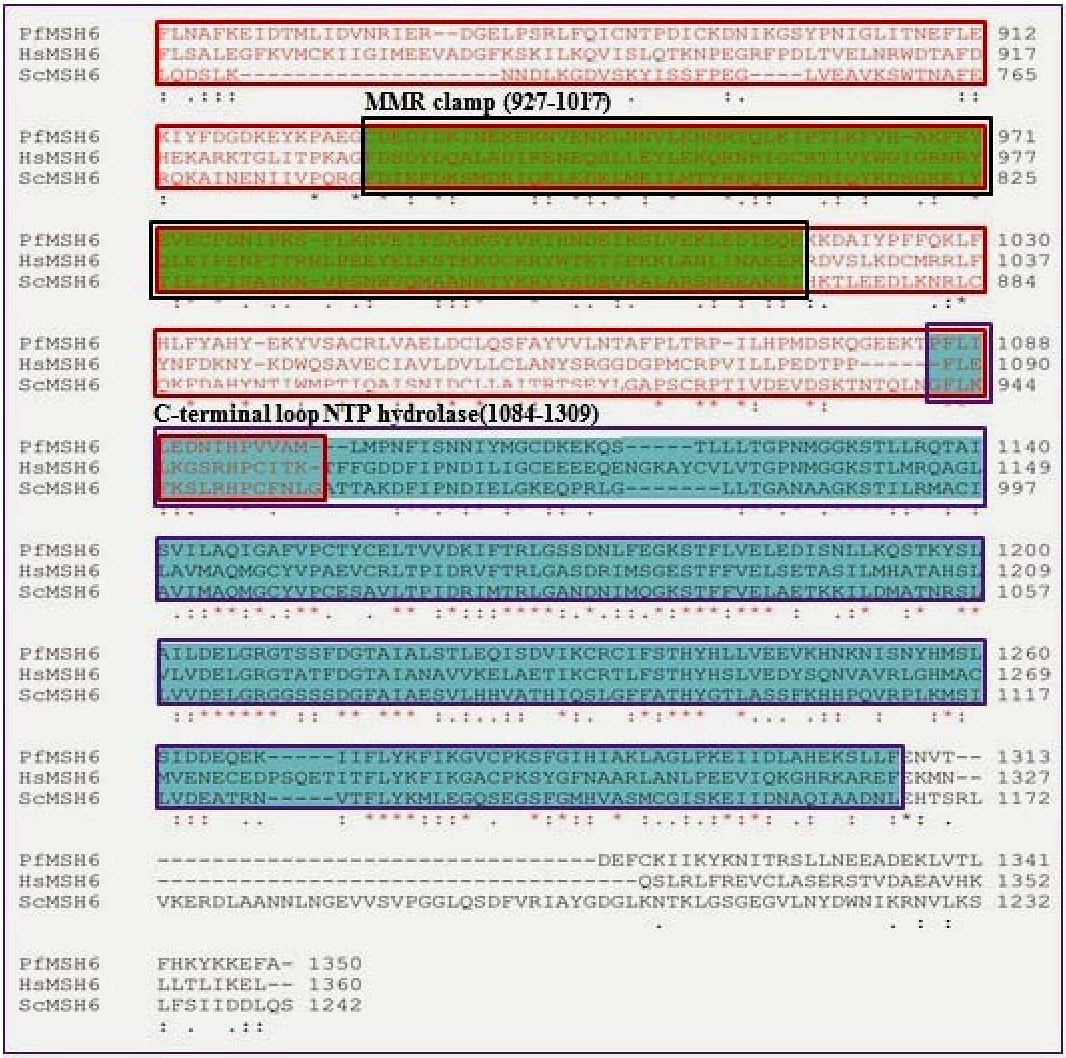
MMR clamp (927-1017)
C-terminal loop NTP hydrolase(1084-1309)
PfMSH6
HsMSH6
ScMSH6

S5-A

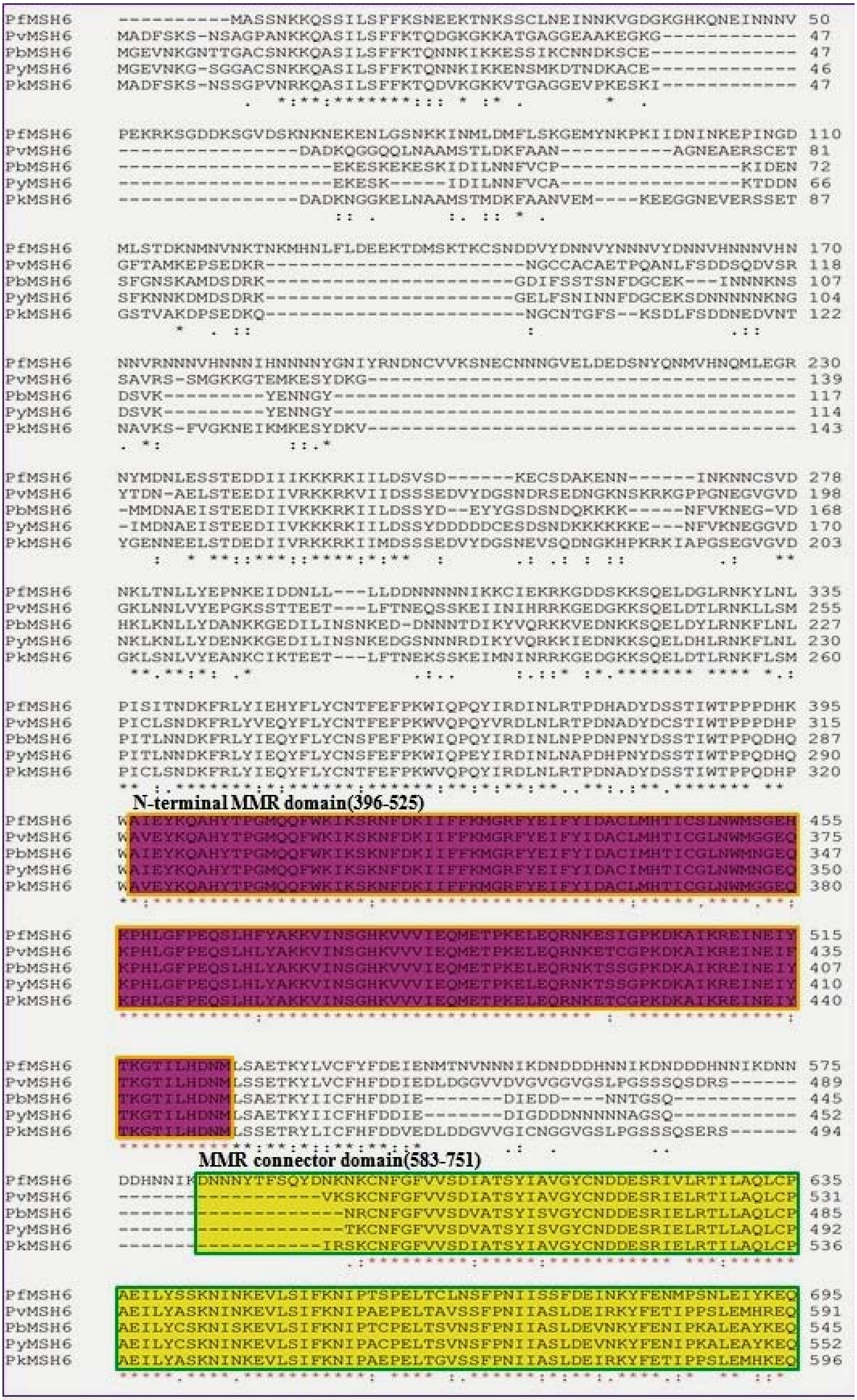

S5-B

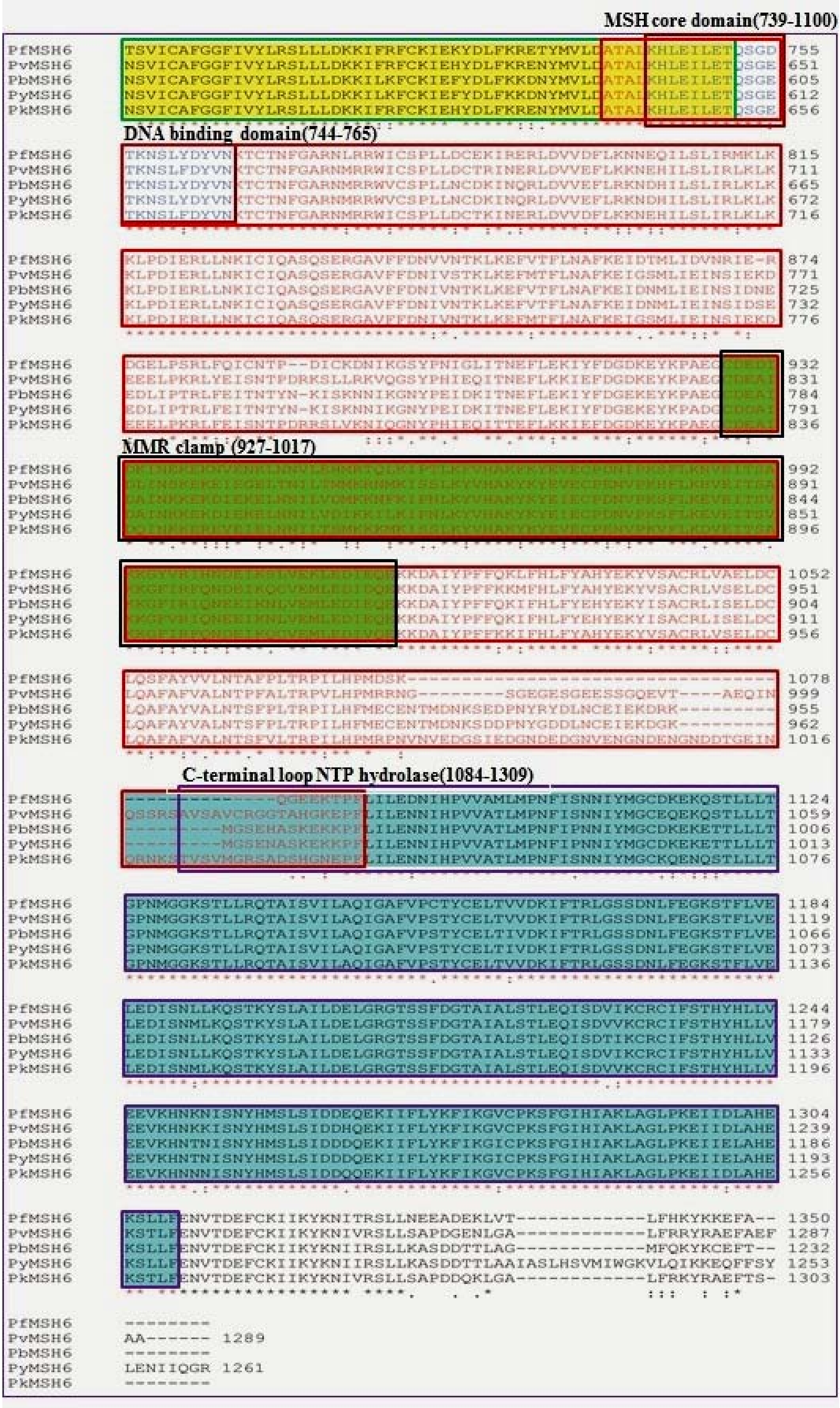
MSH core domain(739-1100)
DNA binding domain(744-765)
MMR clamp (927-1017)
C-terminal loop NTP hydrolase(1084-1309)
PfMSH6
PvMSH6
PbMSH6
PyMSH6
PkMSH6

S5-B
